# Supplementary material for: High-efficiency base editing for nuclear and mitochondrial DNA with an optimized DYW-like deaminase
Source: Mol Ther. 2025 Aug 8;33(11):5611–23. doi: 10.1016/j.ymthe.2025.08.007 (PMC12628173; doi:10.1016/j.ymthe.2025.08.007)
Supplement: Document S1. Figures S1–S11 and Tables S1–S3 [file mmc1.pdf]

YMTHE, Volume *33*

Supplemental Information

High-efficiency base editing for nuclear and mitochondrial DNA with an optimized DYW-like deaminase

Jiyeon Kweon, Soomin Park, Mi Yeon Jeon, Kayeong Lim, Gayoung Jang, An-Hee Jang, Minyoung Lee, Cheong Seok, Chaeyeon Lee, Subin Park, Jiseong Ahn, JiYoon Jang, Naheun Kim, Young Hoon Sung, Daesik Kim, and Yongsub Kim

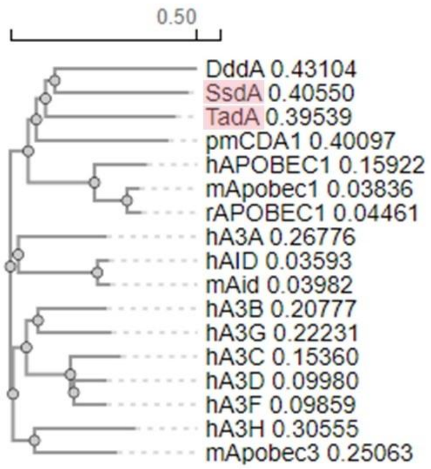

Fig. S1. Phylogenetic tree of representative 18 deaminase domains.

Phylogenetic tree was obtained by T−COFFEE Multiple Sequence Alignment. SsdA and TadA, which served as the basis of rational engineering in this study were highlighted in red.

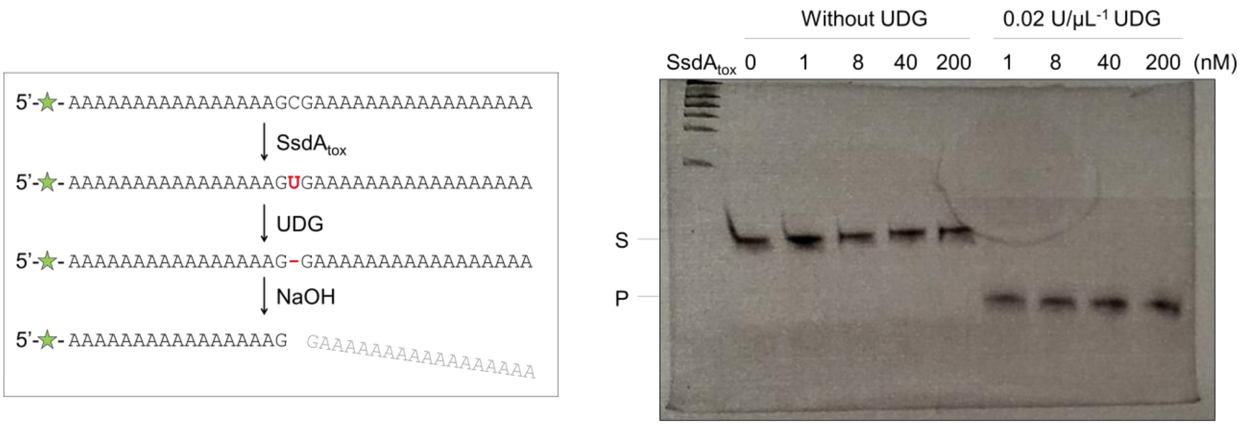

Fig. S2. ***In vitro*** **deaminase assay using SsdA$_{tox}$ protein.**

A 5'-FAM labeled ssDNA containing a single cytosine base nucleotide was used as the substrate for the SsdA$_{tox}$ protein. Cytosine deaminated by the SsdA$_{tox}$ domain is converted to uracil, which is then recognized and cleaved by UDG. NaOH treatment denatures the ssDNA at the cleaved position.

a

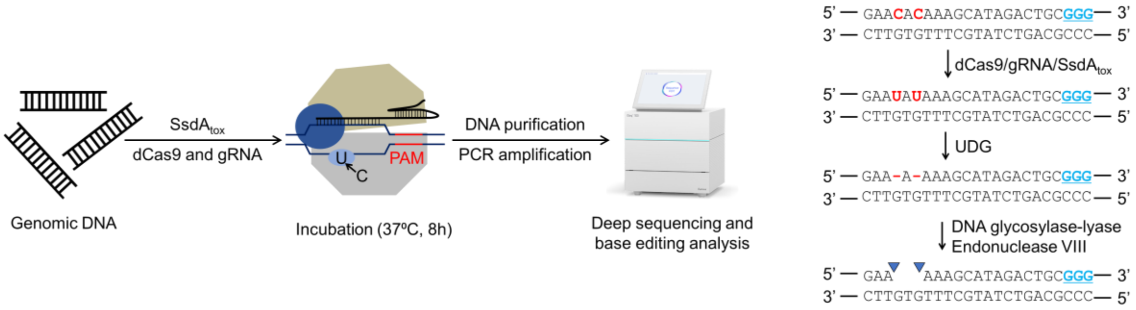

b

RNF2 GTC$_3$ATC$_6$TTAGTC$_{12}$ATTACCTGAGG

USER	-	-	+
RNF2 gRNA	-	+	+
SsdA$_{tox}$/dCas9	-	+	+
C$_3$	0.1	6.5	0.1
C$_6$	0.2	0.0	0.0
C$_{12}$	0.1	10.8	0.0

HEK2 GAAC$_4$AC$_6$AAAGC$_{11}$ATAGACTGCGGG

USER	-	-	+
HEK2 gRNA	-	+	+
SsdA$_{tox}$/dCas9	-	+	+
C$_4$	0.1	18.5	0.0
C$_6$	0.0	18.4	0.0
C$_{11}$	0.1	18.3	0.0

Fig. S3. *In vitro* deaminase assay of SsdA$_{tox}$ with dCas9/gRNA complex.

a Schematic overviews of *in vitro* deaminase assay. Genomic DNA from HEK293T/17 cells were subjected to *in vitro* deaminase assay and cytosine−to−uracil conversion frequencies were measured by targeted deep sequencing. **b** *In vitro* deaminase assay utilizing the SSdA$_{tox}$ domain showed C−to−T conversion efficiencies at the RNF2 (C3, C6, and C12) and HEK2 (C4, C6, and C11) target sites measured by targeted deep sequencing. The maximum conversion efficiency reached up to 50%, limited to editing of cytosine in the target strand only.

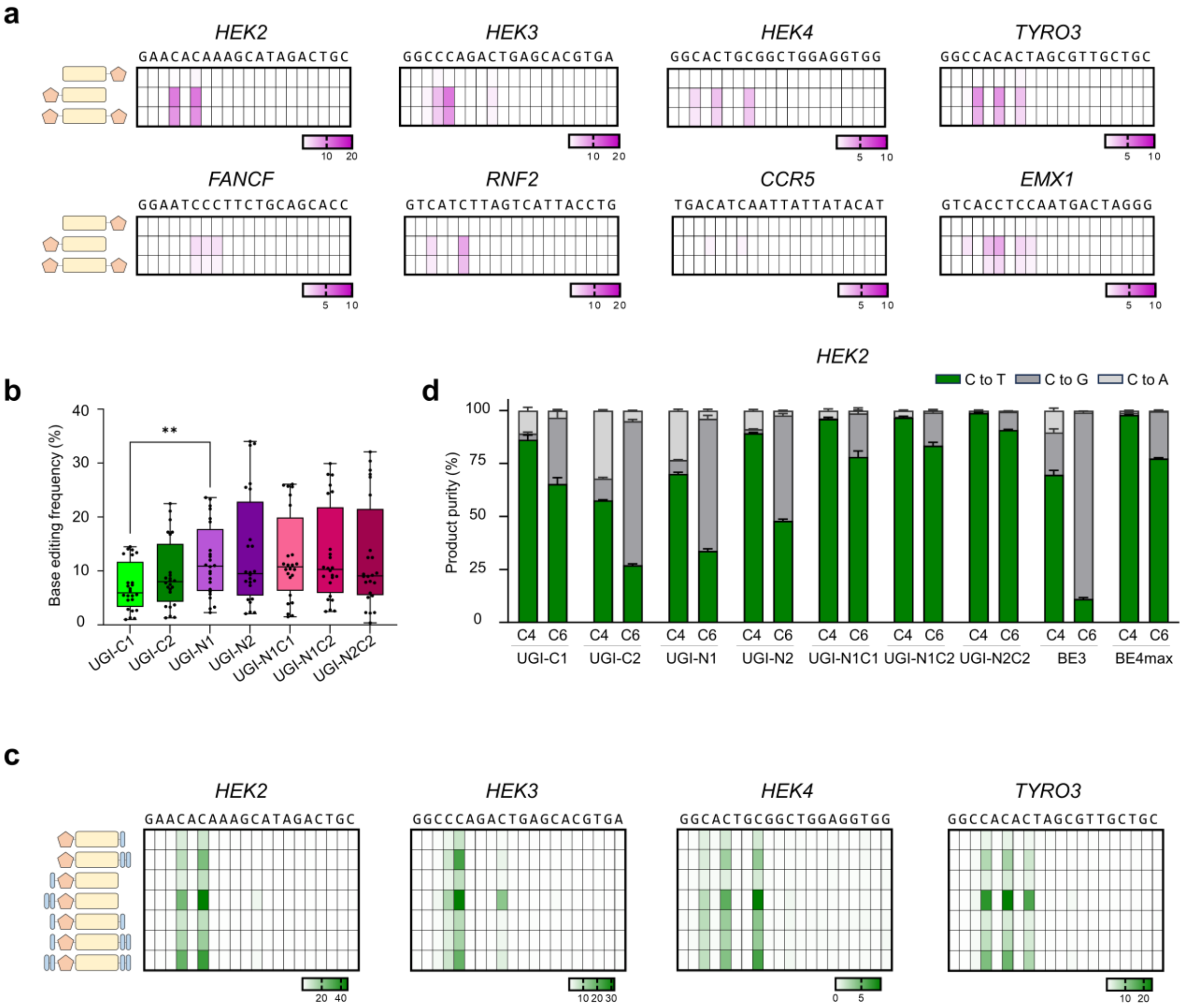

Fig. S4. Developments of $SsdA_{tox}$–based cytosine base editors.

a Base editing frequency of each construct in HEK293T/17 *UNG* KO cells across eight target sites were described in heatmap. **b** Base editing frequencies of each construct across 8 target sites in *UNG* KO HEK293T/17 cells were depicted in box-whisker plot. Dots represent the three independent biological replicates of each 8 target sites. ** P=0.0056 by unpaired t-test. **c** Base editing frequency of each construct in wild-type HEK293T/17 cells across four target sites were described in heatmap. **d** Product purity comparison of each construct at HEK2 C4 and C6 target site in wild-type HEK293T/17 cells. Data are represented as mean of three independent biological replicates (n=3) samples, with error bars representing the SEM.

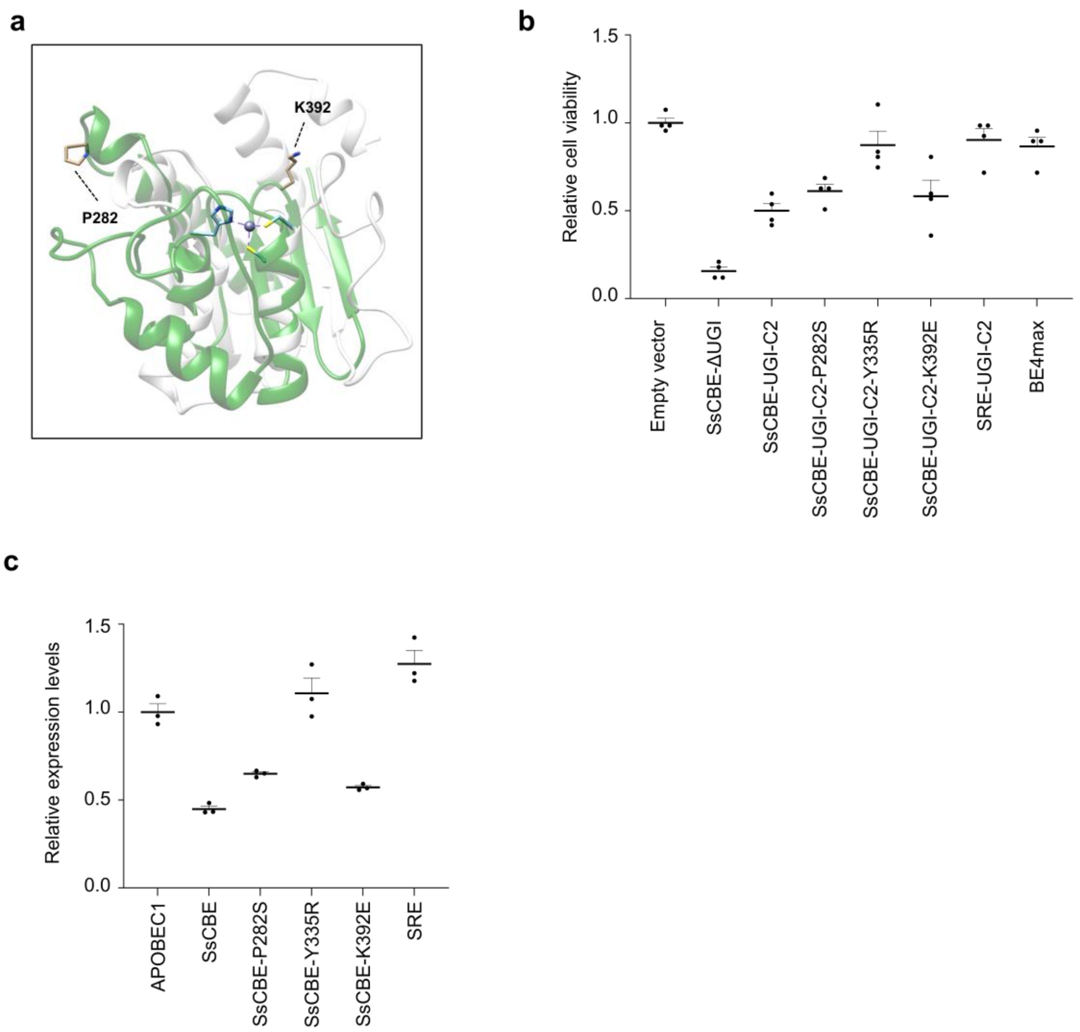

Fig. S5. Cytotoxicity and expression of engineered SsdAtox variants.

a Structural alignment of TadA and $SsdA_{tox}$ domain highlighting two candidate residues for engineering: P282 and K392. **b, c** To measure cell viability, each $SsdA_{tox}$ variant was transfected into HEK293T/17 cells, and the luminescent assay was conducted 72h after transfection. Relative cell viabilities were calculated by dividing the Relative Light Units (RLUs) of cells transfected with each variant by the RLUs of cells transfected with the empty vector. To compare expression level, the P2A-mcherry fused each deaminase domain was transfected into HEK293T/17 cells and FACS analysis was performed. The relative expression levels were calculated by dividing the percentage of PerCP−Cy5−5−A positive cells transfected with each variant by the percentage of PerCP−Cy5−5−A positive cells transfected with the rAPOBEC1−P2A−mcherry construct. The transfection was conducted in three independent biological replicates (n=3).

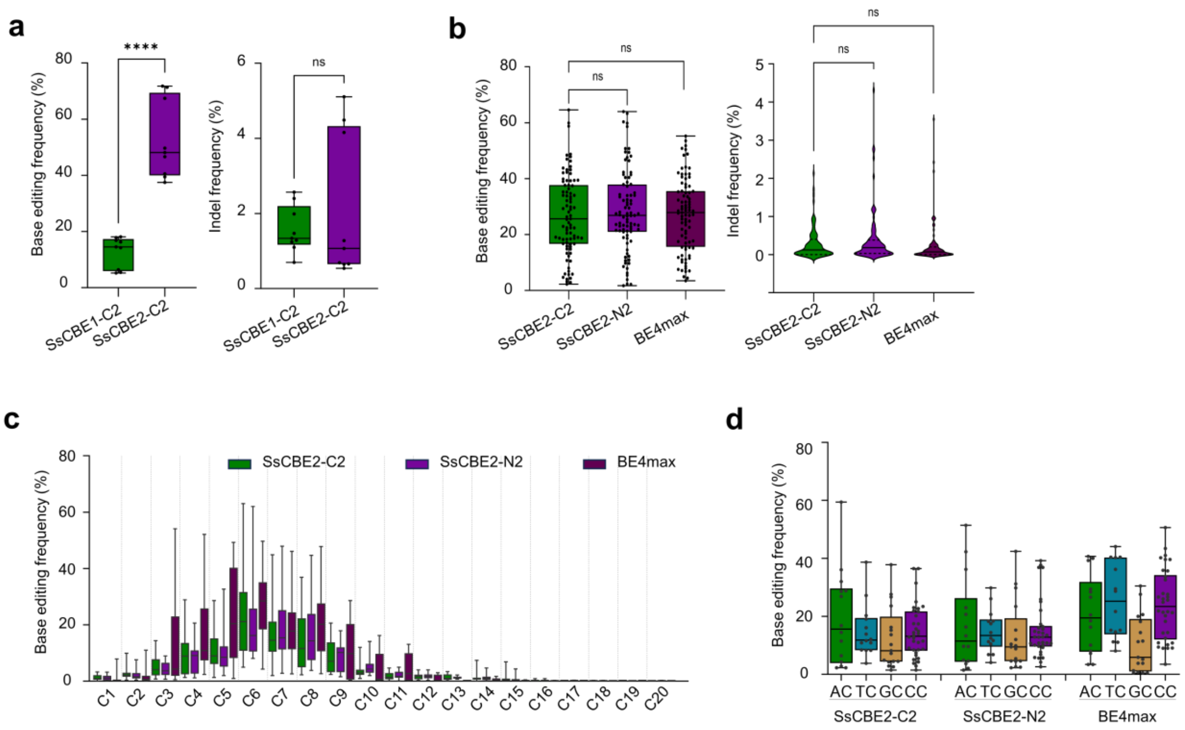

Fig. S6. Rational engineering of SsdA$_{tox}$ domain.

a Comparison of base editing and indel frequency of SsCBE1−C2 and SsCBE2−C2 across three target sites in HEK293T/17 cells. **** P<0.0001 by unpaired t-test. **b** Comparison of base editing and indel frequency of SsCBE2−C2, SsCBE2−N2, and BE4max across 29 endogenous target sites in HEK293T/17 cells. Statistical analysis performed using unpaired t-test. **c, d** Base editing window and context analysis of three constructs across 29 endogenous target sites in HEK293T/17 cells.

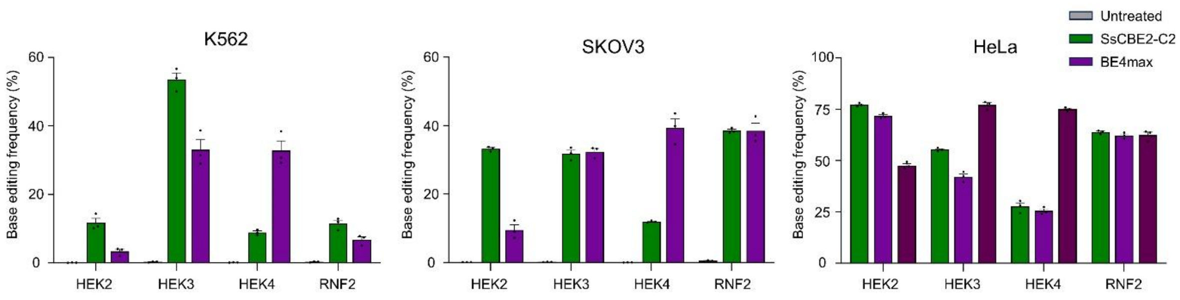

Fig. S7. Base editing in other three cell lines.

SsCBE2-C2 and BE4max were transfected in three different cell line and base editing frequencies were measured by targeted deep sequencing across 4 target sites. Data are presented as mean, with error bars representing SEM of three independent biological replicates (n=3).

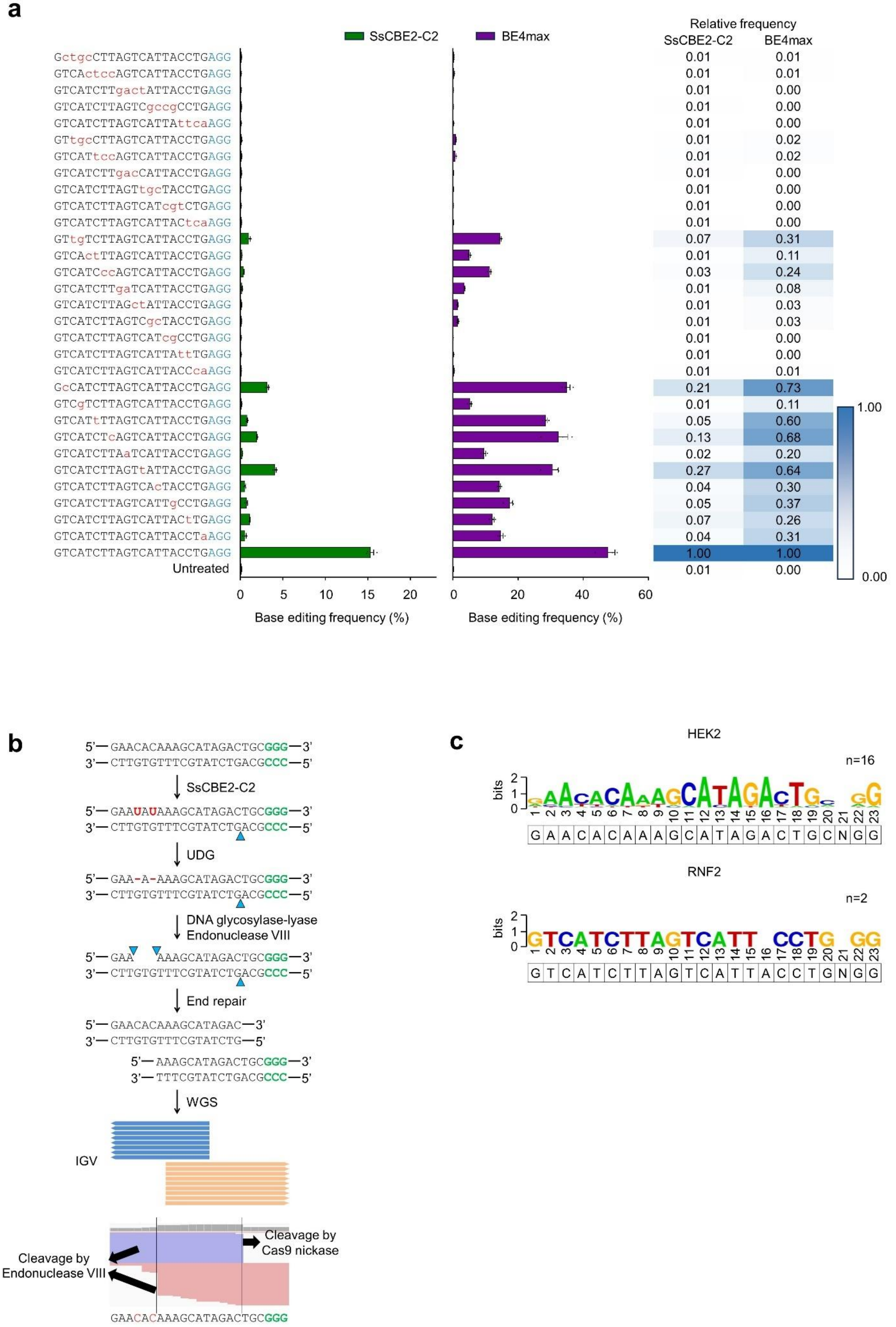
a
SsCBE2-C2
BE4max
Relative frequency
SsCBE2-C2 BE4max
GctgcCTTAGTCATTACCTGAGG 0.01 0.01
GTCActccAGTCATTACCTGAGG 0.01 0.01
GTCATCTTgactATTACCTGAGG 0.01 0.00
GTCATCTTAGTCgccgCCTGAGG 0.01 0.00
GTCATCTTAGTCATTAttcaAGG 0.01 0.00
GTtgcCTTAGTCATTACCTGAGG 0.01 0.02
GTCATtccAGTCATTACCTGAGG 0.01 0.02
GTCATCTTgacCATTACCTGAGG 0.01 0.00
GTCATCTTAGTtgcTACCTGAGG 0.01 0.00
GTCATCTTAGTCATcgtCTGAGG 0.01 0.00
GTCATCTTAGTCATTACtcaAGG 0.01 0.00
GTtgTCTTAGTCATTACCTGAGG 0.07 0.31
GTCActTTAGTCATTACCTGAGG 0.01 0.11
GTCATCccAGTCATTACCTGAGG 0.03 0.24
GTCATCTTgaTCATTACCTGAGG 0.01 0.08
GTCATCTTAGctATTACCTGAGG 0.01 0.03
GTCATCTTAGTCgcTACCTGAGG 0.01 0.03
GTCATCTTAGTCATcgCCTGAGG 0.01 0.00
GTCATCTTAGTCATTAttTGAGG 0.01 0.00
GTCATCTTAGTCATTACCcaAGG 0.01 0.01
GcCATCTTAGTCATTACCTGAGG 0.21 0.73
GTCgTCTTAGTCATTACCTGAGG 0.01 0.11
GTCATtTTAGTCATTACCTGAGG 0.05 0.60
GTCATCTcAGTCATTACCTGAGG 0.13 0.68
GTCATCTTAaTCATTACCTGAGG 0.02 0.20
GTCATCTTAGTtATTACCTGAGG 0.27 0.64
GTCATCTTAGTCAcTACCTGAGG 0.04 0.30
GTCATCTTAGTCATTgCCTGAGG 0.05 0.37
GTCATCTTAGTCATTACtTGAGG 0.07 0.26
GTCATCTTAGTCATTACCTaAGG 0.04 0.31
GTCATCTTAGTCATTACCTGAGG 1.00 1.00
Untreated 0.01 0.00
1.00
0.00
0 5 10 15 20
Base editing frequency (%)
0 20 40 60
Base editing frequency (%)
b
5'— GAACACAAAGCATAGACTGCGGG —3'
3'— CTTGTGTTTCGTATCTGACGCCC —5'
SsCBE2-C2
5'— GAAUAUAAAGCATAGACTGCGGG —3'
3'— CTTGTGTTTCGTATCTGACGCCC —5'
UDG
5'— GAA-A-AAAGCATAGACTGCGGG —3'
3'— CTTGTGTTTCGTATCTGACGCCC —5'
DNA glycosylase-lyase
Endonuclease VIII
5'— GAA AAAGCATAGACTGCGGG —3'
3'— CTTGTGTTTCGTATCTGACGCCC —5'
End repair
5'— GAACACAAAGCATAGAC —3'
3'— CTTGTGTTTCGTATCTG —5'
5'— AAAGCATAGACTGCGGG —3'
3'— TTTCGTATCTGACGCCC —5'
WGS
IGV
Cleavage by
Cas9 nickase
Cleavage by
Endonuclease VIII
GAACACAAAGCATAGACTGCGGG
c
HEK2
n=16
bits
G A A C A C A A A G C A T A G A C T G C N G G
RNF2
n=2
bits
G T C A T C T T A G T C A T T A C C T G N G G

Fig. S8. Analysis of gRNA dependent DNA off-target effects of SsCBE2.

a Tolerance evaluation of SsCBE2−C2 and BE4max for mismatched sgRNAs with one to four nucleotides mismatches from the RNF2 site in HEK293T/17 cells. PAM sequences are indicated in blue and mismatched bases are indicated in red. Relative frequencies were calculated by dividing base editing frequencies obtained with mismatched sgRNAs by the mean base editing frequency of the matched sgRNA. Data are presented as mean, with error bars representing SEM of three independent biological replicate (n=3). **b** Overviews of Digenome−seq using SsCBE2−C2 at HEK2 target site. SsCBE−C2 catalyzes C−to−U conversion and the uracil−containing sites were cleaved by USER enzyme, a mixture of *E.coli* uracil DNA glycosylase (UDG) and DNA glycosylase−lyase endonuclease VIII. Arrows indicate the positions of phosphodiester bonds cleaved by the SpCas9(D10A) nickase and USER. IGV image shows straight alignments of sequence reads at HEK2 on-target site. **c** Nucleotide sequences captured by Digenome−seq were compared and sequence logos were obtained using WebLogo.

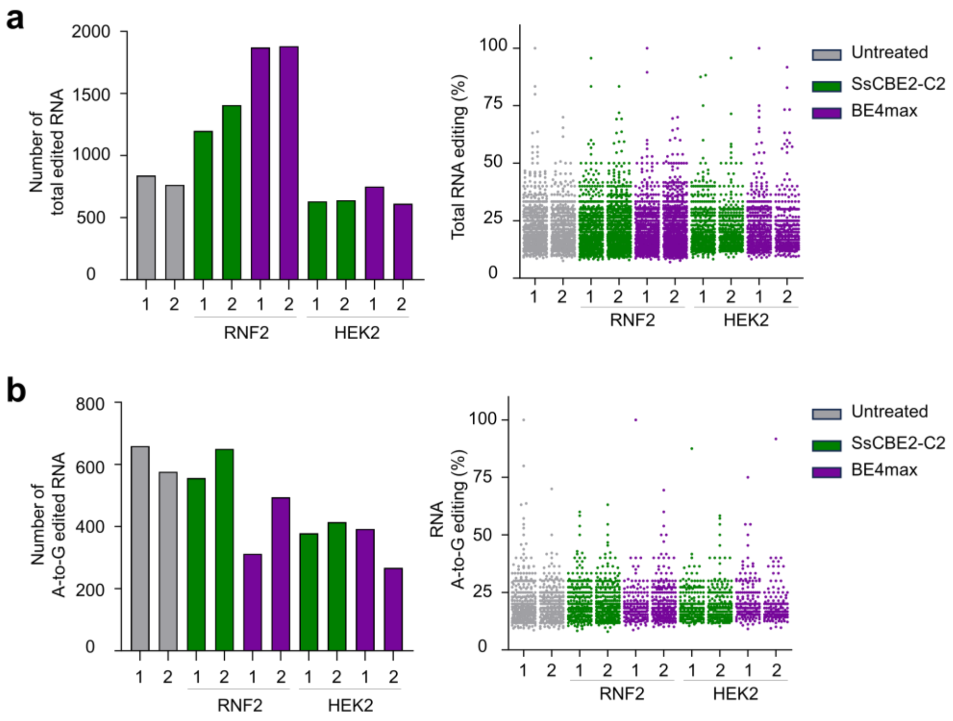

Fig. S9. Analysis of Cas9−independent RNA off−target effects of SsCBE2.

Cas9−independent RNA off−target deamination of SsCBE2−C2 and BE4max in HEK293T/17 cells. Transcriptome sequencing was used to determine the number and frequency of total RNA editing, **a**, and of A−to−G RNA editing, **b**.

a

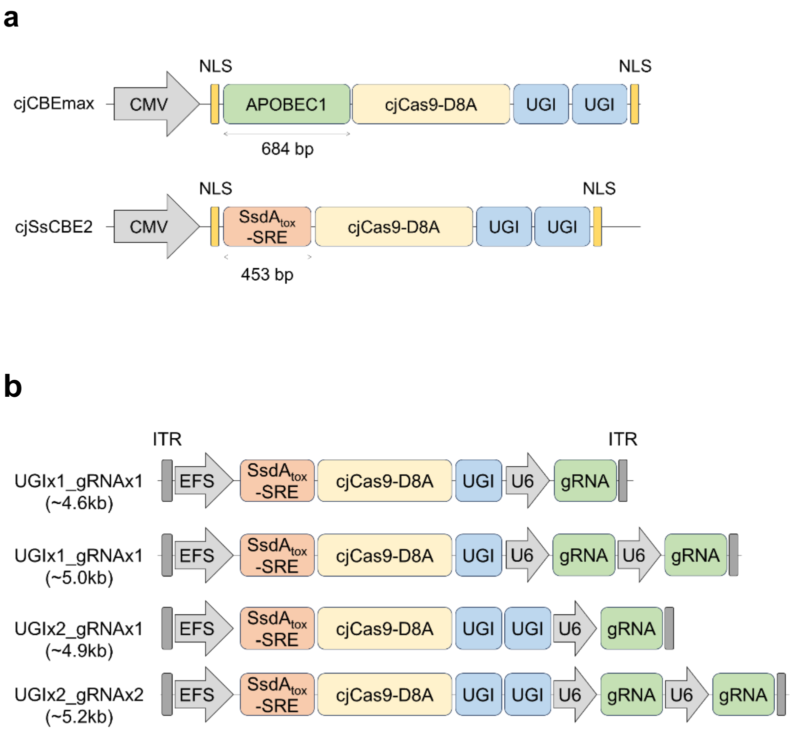

Fig. S10. Architectures of cjCas9-based cytosine base editors.

a Schematic overview of cjSsCBE2 and cjCBEmax. **b** Schematic overviews of single AAV vectors encoding various cjSsCBE2 variants and gRNA. The length between ITR is described in each construct.

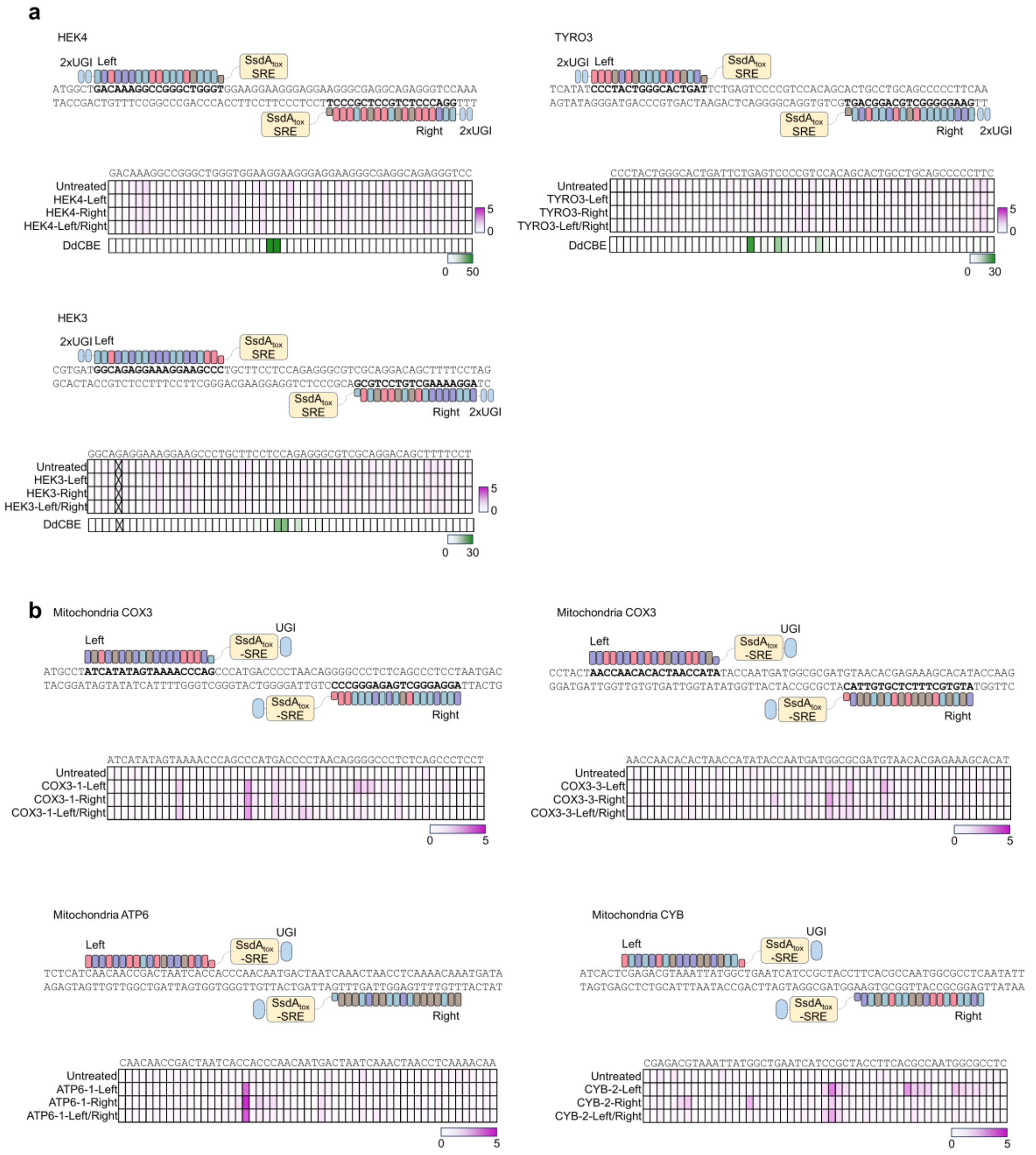

Fig. S11. Base editing using TALE-SRE.

a Base editing frequency of TALE−SRE targeting nucleus genome. Monomer or dimer forms of TALE−SRE were transfected in HEK293T/17 cells and DdCBE was used as positive controls. Transfection was conducted in biological triplicate and base editing frequencies were described in heatmaps. **b** Base editing frequencies induced by TALE–SRE across the target region, including TALE-binding sites and the spacer. Transfection was conducted in biological triplicate and base editing frequencies were described in heatmaps.

Table S1. Identified potential off-target sites by Digenome-seq.

HEK2	Chr.	Location	DNA cleavage score	DNA seq at a cleavage site	Bulge
HEK2_ON	chr.5	87240613	14.2	GAACACAAAGCATAGACTGCGGG	X
HEK2_OT1	chr.15	93557679	7.5	GAACACA-tGCATAGACTGCTAG	O
HEK2_OT2	chr.4	90522183	6.7	GAACACAAtGCATAGAtTGCCGG	X
HEK2_OT3	chr.2	19844956	1.3	aActcCAAAGCATAtACTGCTGG	X
HEK2_OT4	chr.13	55564918	1.2	acACACAAAGCAT-GACTGCAGG	X
HEK2_OT5	chr.1	167742859	0.7	aAACACAgAGCAcAGACTGCTGA	X
HEK2_OT6	chr.19	35505485	0.6	GAACAC-AAGCAcAGACTGaAGG	O
HEK2_OT7	chr.1	36097072	0.4	GtAaACAAAGCATAGACTGaGGG	X
HEK2_OT8	chr.2	192248363	0.4	GAACACAtA-CATAGACaGCTGG	X
HEK2_OT9	chr.11	128508576	0.3	GAAttCAAAGCATAGAtTGCAGG	X
HEK2_OT10	chr.1	77190607	0.3	tcACACAAAcCATAGACTGaGGG	X
HEK2_OT11	chr.4	135329594	0.3	tAACAaAtAGCATAGACTGtGTG	X
HEK2_OT12	chr.8	97317606	0.3	GAACACAgtaCATAGACTGgCAG	X
HEK2_OT13	chr.9	290167	0.2	aAACAtAAAGaATAGACTGCAAG	X
HEK2_OT14	chr.4	53536209	0.2	GAAtACtAAGCATAGACTcCAGG	X
HEK2_OT15	chr.19	28824655	0.2	GAACtCAAAGCATAGAaTaaTGG	X
**RNF2**					
RNF2_ON	chr.1	185056773	3.1	GTCATCTTAGTCATTACCTGAGG	X
RNF2_OT2	chr.10	75832488	0.5	GcCATCTTAGTCATT-CCTGGGG	O

Table S2. Nucleotide sequences of target sites and PCR primers.

Target sites	Spacer sequences	PAM	PCR-F	PCR-R
AAVS1	GCTGACTCAGAGACCCTGAG	TGG	GGCCCAGACTAGCCCAGTTGT	CCACCTGCCTTGGCCTCTCA
CCR5	TGACATCAATTATTATACAT	CGG	GAGGGCAACTAAATACATTCTAGGAC	CCAAAGATGAACACCAGTGA
CUL3	GTAAACCTGGAATAACACGA	TGG	TTGGGAGCACTTCCAGGTTCACT	CTGCACTCCAGCCTTGGTGACAG
EMX1	GTCACCTCCAATGACTAGGG	TGG	GGACAAAGTACAAACGGCAGA	AGTGGCCAGAGTCCAGCTT
FANCF	GGAATCCCTTCTGCAGCACC	TGG	ATGGATGTGGCGCAGGTAG	AGCATTGCAGAGAGGCGTAT
HEK2	GAACACAAAGCATAGACTGC	GGG	AGACCTGGCTGAGCTAACTG	TCCAGCCCCATCTGTCAAAC
HEK3	GGCCCAGACTGAGCACGTGA	TGG	GCATGCATTTGTAGGCTTGA	CCCAGCCAAACTTGTCAAC
HEK4	GGCACTGCGGCTGGAGGTGG	GGG	CTCCCTTCAAGATGGCTGAC	AACGGAGACACACACACAGG
RNF2	GTCATCTTAGTCATTACCTG	AGG	ATTTCCAGCAATGTCTCAGG	GCCAACATACAGAAGTCAGGAA
TYRO3	GGCCACACTAGCGTTGCTGC	TGG	TCCCTACTGGGCACTGATTC	TCCCTGTCAACAAAGTGCTG
Site-1	CCAGCCCGCTGGCCCTGTAA	AGG	AGACCTGGCTGAGCTAACTG	TCCAGCCCCATCTGTCAAAC
Site-2	GCTGGCCCTGTAAAGGAAAC	TGG	AGACCTGGCTGAGCTAACTG	TCCAGCCCCATCTGTCAAAC
Site-3	GTTTCCTTTACAGGGCCAGC	GGG	AGACCTGGCTGAGCTAACTG	TCCAGCCCCATCTGTCAAAC
Site-4	GCACTTGTTTGCAGCTATTC	AGG	AGACCTGGCTGAGCTAACTG	TCCAGCCCCATCTGTCAAAC
Site-5	CTGCTTCTCCAGCCCTGGCC	TGG	GCATGCATTTGTAGGCTTGA	CCCAGCCAAACTTGTCAAC
Site-6	CCCTGGCCTGGGTCAATCCT	TGG	GCATGCATTTGTAGGCTTGA	CCCAGCCAAACTTGTCAAC
Site-7	GGAAGCCCTGCTTCCTCCAG	AGG	GCATGCATTTGTAGGCTTGA	CCCAGCCAAACTTGTCAAC
Site-8	CTTCCTCCAGAGGGCGTCGC	AGG	GCATGCATTTGTAGGCTTGA	CCCAGCCAAACTTGTCAAC
Site-9	CAGGACAGCTTTTCCTAGAC	AGG	GCATGCATTTGTAGGCTTGA	CCCAGCCAAACTTGTCAAC
Site-10	CAGCTCCTGCACCGGGATAC	TGG	GCATGCATTTGTAGGCTTGA	CCCAGCCAAACTTGTCAAC
Site-11	GGGGCACCGCGGCGCCCCGG	TGG	CTCCCTTCAAGATGGCTGAC	AACGGAGACACACACACAGG
Site-12	GCGGCGCCCCGGTGGCACTG	CGG	CTCCCTTCAAGATGGCTGAC	AACGGAGACACACACACAGG
Site-13	CGCCCCGGTGGCACTGCGGC	TGG	CTCCCTTCAAGATGGCTGAC	AACGGAGACACACACACAGG
Site-14	TCCCTTCCTTCCACCCAGCC	CGG	CTCCCTTCAAGATGGCTGAC	AACGGAGACACACACACAGG
Site-15	CCCTGCCTGTCATCCTGCTT	TGG	CTCCCTTCAAGATGGCTGAC	AACGGAGACACACACACAGG
Site-16	GCAGTGCCACCGGGGCGCCG	CGG	CTCCCTTCAAGATGGCTGAC	AACGGAGACACACACACAGG
Site-17	CTCCAGCCGCAGTGCCACCG	GGG	CTCCCTTCAAGATGGCTGAC	AACGGAGACACACACACAGG
Site-18	ACCTCCAGCCGCAGTGCCAC	CGG	CTCCCTTCAAGATGGCTGAC	AACGGAGACACACACACAGG
Site-19	TACACGTCTCATATGCCCCT	TGG	ATTTCCAGCAATGTCTCAGG	GCCAACATACAGAAGTCAGGAA
Site-20	TCAACCATTAAGCAAAACAT	GGG	ATTTCCAGCAATGTCTCAGG	GCCAACATACAGAAGTCAGGAA
Rloop-site-1	GTGGTAGACAGCATGTGTCCTA	AAGGG	TCCTGCAGTCTCCTGCTTCT	ACCAACATACATGCCCCTTT
Rloop-site-2	ATTTACAGCCTGGCCTTTGGGG	TCGGG	GACATTTCCACCGCAAAATG	CGGTGGGAGATCTGGTTTC
Rloop-site-3	GTGTCAGGTAATGTGCTAAACA	GAGAG	TGCTCCAGATTTCCCTTCAT	GGCATCCAGAGACATGGTTT
Rloop-site-4	TCTGCTTCTCCAGCCCTGGC	CTGGG	AAACGCCCATGCAATTAGTC	CAGGAGCTGCACATACTAGCC
Rloop-site-5	GATGTTCCAATCAGTACGCA	GAGAG	GAAAAGCGATCCAGGTGCT	GGCTTTTAAGTTGCCCAGAG
EPAS	CAGGACAGCAGGGGCTCCTTGT	AGCCACAC	AAGCCTTGGAGGGTTTCATT	GTGGCTAGCACCTTCCACTC
HIF	GCTATTCACCAAAGTTGAATCA	GAAGATAC	CCCTTCCCCTCACTGTATCA	GGCCAGCAAAGTTAAAGCAT
ANGPT2	GCTGTGCAGAGGGACGCGCCGC	TCGAATAC	ATGGGTCCTGCAGCTACACT	TTCCATGATGTTCTCCAGCA
HPD-1	TTTTCCACCCGTAGTATGGGGA	CACCACAC	GGAAGTAGGGGGTCCATGA	ACGCATCTGGTTAGGGTCAG
HPD-2	TTCCACCCGTAGTATGGGGACA	CCACACAC	GGAAGTAGGGGGTCCATGA	ACGCATCTGGTTAGGGTCAG
mTyr	ACCTCAGTTCCCCTTCAAAG	GGG	AACCCATGAAGTTGCCTGAG	TTGTTGGCAAAAGAATGCTG

Table S3. Target sequences and PCR sequences for TALE-SRE.

Target sites	Target sequence	PCR-F	PCR-R
ND1-Left	AGCCGTTTACTCAATCCTC	GCTCTCACCATCGCTCTTCT	TGATGGCTAGGGTGACTTCAT
ND1-Right	CAGGGCGTAGTTTGAGTTT	GCTCTCACCATCGCTCTTCT	TGATGGCTAGGGTGACTTCAT
ATP6-1-Left	CAACAACCGACTAATCACC	CCCTCTATTGATCCCCACCT	GATGGCCATGGCTAGGTTTA
ATP6-2-Right	TTGTTTTGAGGTTAGTTTG	CCCTCTATTGATCCCCACCT	GATGGCCATGGCTAGGTTTA
ATP6-2-Left	AACCATACACAACACTAAA	CCCTCTATTGATCCCCACCT	GATGGCCATGGCTAGGTTTA
ATP6-2-Right	GATTAAGGATACTAGTATA	CCCTCTATTGATCCCCACCT	GATGGCCATGGCTAGGTTTA
COX3-1-Left	ATCATATAGTAAAACCCAG	CAACACATAATGACCCACCAA	GAAGGCCTTTTTGGACAGGT
COX3-1-Right	AGGAGGGCTGAGAGGGCCC	CAACACATAATGACCCACCAA	GAAGGCCTTTTTGGACAGGT
COX3-2-Left	CCTAATGACCTCCGGCCTA	CAACACATAATGACCCACCAA	GAAGGCCTTTTTGGACAGGT
COX3-2-Right	GAGGAGCGTTATGGAGTGG	CAACACATAATGACCCACCAA	GAAGGCCTTTTTGGACAGGT
COX3-3-Left	AACCAACACACTAACCATA	CAACACATAATGACCCACCAA	GAAGGCCTTTTTGGACAGGT
COX3-3-Right	ATGTGCTTTCTCGTGTTAC	CAACACATAATGACCCACCAA	GAAGGCCTTTTTGGACAGGT
CYB-1-Left	CCAACATCTCCGCAT	AACCACTCATTCATCGACCTC	CGCCCGATGTGTAGGAAG
CYB-1-Right	CAGGCAGGCGCCAAGGAGT	AACCACTCATTCATCGACCTC	CGCCCGATGTGTAGGAAG
CYB-2-Left	CGAGACGTAAATTATGGC	AACCACTCATTCATCGACCTC	CGCCCGATGTGTAGGAAG
CYB-2-Right	GAGGCGCCATTGGCGTGAA	AACCACTCATTCATCGACCTC	CGCCCGATGTGTAGGAAG
HEK4-Left	GACAAAGGCCGGGCTGGGT	CTCCCTTCAAGATGGCTGAC	AACGGAGACACACACACAGG
HEK4-Right	GGACCCTCTGCCTCGCCCT	CTCCCTTCAAGATGGCTGAC	AACGGAGACACACACACAGG
TYRO3-Left	CCCTACTGGGCACTGAT	TCCCTACTGGGCACTGATTC	TCCCTGTCAACAAAGTGCTG
TYRO3-Right	GAAGGGGGCTGCAGGCAGT	TCCCTACTGGGCACTGATTC	TCCCTGTCAACAAAGTGCTG
HEK3-Left	GGCAGAGGAAAGGAAGCCC	GCATGCATTTGTAGGCTTGA	CCCAGCCAAACTTGTCAAC
HEK3-Right	AGGAAAAGCTGTCCTGCG	GCATGCATTTGTAGGCTTGA	CCCAGCCAAACTTGTCAAC
