# Supplementary material for: High-efficiency base editing for nuclear and mitochondrial DNA with an optimized DYW-like deaminase
Source: Mol Ther. 2025 Aug 8;33(11):5611–23. doi: 10.1016/j.ymthe.2025.08.007 (PMC12628173; doi:10.1016/j.ymthe.2025.08.007)
Supplement: Document S2. Article plus supplemental information [file mmc2.pdf]

Molecular Therapy
Original Article

High-efficiency base editing for nuclear and mitochondrial DNA with an optimized DYW-like deaminase

Jiyeon Kweon,[1,2,7] Soomin Park,[3,7] Mi Yeon Jeon,[1,4] Kayeong Lim,[5,6] Gayoung Jang,[1,2] An-Hee Jang,[1,2] Minyoung Lee,[3] Cheong Seok,[3] Chaeyeon Lee,[1,2] Subin Park,[3] Jiseong Ahn,[1,2] JiYoon Jang,[1,2] Naheun Kim,[1,2] Young Hoon Sung,[1,4] Daesik Kim,[3] and Yongsub Kim[1,2]

[1]Department of Cell and Genetic Engineering, BK21 Project, Asan Medical Center, University of Ulsan College of Medicine, Seoul 05505, Republic of Korea; [2]Stem Cell Immunomodulation Research Center, University of Ulsan College of Medicine, Seoul 05505, Republic of Korea; [3]Department of Precision Medicine, Sungkyunkwan University School of Medicine, Suwon 16419, Republic of Korea; [4]Convergence Medicine Research Center, Asan Institute for Life Sciences, Asan Medical Center, Seoul 05505, Republic of Korea; [5]Brain Science Institute, Korea Institute of Science and Technology (KIST), Seoul 02792, Republic of Korea; [6]Division of Bio-Medical Science & Technology, KIST School, Korea University of Science and Technology, Seoul 02792, Republic of Korea

CRISPR-based cytosine base editors enable precise genome editing without inducing double-stranded DNA breaks yet traditionally depend on a limited selection of deaminases from the APOBEC/AID or TadA families. Here, we present SsCBE, a CRISPR-based cytosine base editor utilizing SsdA$_{tox}$, a DYW-like deaminase derived from the toxin of *Pseudomonas syringae*. Strategic engineering of SsdA$_{tox}$ has led to remarkable improvements in the base editing efficiency (by up to 8.4-fold) and specificity for SsCBE, while concurrently reducing cytotoxicity. Exhibiting exceptional versatility, SsCBE was delivered and efficiently applied using diverse delivery methods, including engineered virus-like particles. Its application has enabled targeted cytosine base editing in mouse zygotes and pioneering edits in mitochondrial DNA. SsCBE expands the genome editing toolbox by introducing a distinct deaminase scaffold with broad utility for both basic research and potential therapeutic applications.

INTRODUCTION

Base editing, a cutting-edge development in the field of genome engineering, represents a significant leap beyond traditional genome editing tools. It allows for the precise and targeted alteration of nucleotide sequences without the need for DNA double-stranded breaks or donor templates.[1] Central to the mechanism of base editors (BEs) are its two main components: DNA-binding modules, such as the CRISPR-Cas system, transcription-activator-like effectors (TALEs), and zinc-finger proteins (ZFPs), and the deaminase enzymes that facilitate direct nucleotide conversion. CRISPR-based base editing exemplifies this evolution, employing a fusion of Cas9 with single-stranded DNA (ssDNA) targeting deaminases, such as those from the AID/APOBEC family or engineered TadA variants.[2–4] The Cas9-guide RNA (gRNA) complex binds the targeted DNA sequence and forms an R-loop, exposing ssDNA. The exposed strand is then edited by the deaminase, converting specific nucleotides with high precision. An alternative strategy incorporates TALEs or ZFPs with double-stranded DNA (dsDNA)-targeting deaminases like DddA$_{tox}$ derived from the SCP1.201 family, further diversifying the base editing toolkit and expanding its potential applications for organellar base editing.[5–8]

Despite these advancements, the range of deaminases currently employed in base editing is somewhat limited. Predominantly, members of the AID/APOBEC family are utilized in cytosine base editors (CBEs), TadA variants are employed in both CBEs and adenine base editors (ABEs), and DddA variants are used in dsDNA-targeting CBEs.[9–13] In this study, we aim to further expand the toolkit of base editing by introducing a novel, compact, and highly efficient deaminase from the DYW-like deaminase, integrated with Cas9 and TALE systems. We fused nCas9 with SsdA$_{tox}$, known as a bacterial toxin, derived from *Pseudomonas syringae*, enabling targeted cytosine base editing and named SsCBE (SsdA$_{tox}$-derived CBE).[14] Through rational engineering of SsdA$_{tox}$, we successfully improved the base editing efficiency of SsCBE comparable to conventional

Received 14 April 2025; accepted 5 August 2025;
https://doi.org/10.1016/j.ymthe.2025.08.007.

[7]These authors contributed equally

Correspondence: Jiyeon Kweon, Department of Cell and Genetic Engineering, BK21 Project, Asan Medical Institute of Convergence Science and Technology, Asan Medical Center, University of Ulsan College of Medicine, Seoul 05505, Republic of Korea.
E-mail: jiyeon1015@gmail.com
Correspondence: Daesik Kim, Department of Precision Medicine, Sungkyunkwan University School of Medicine, Suwon 16419, Republic of Korea.
E-mail: dskim89@skku.edu
Correspondence: Yongsub Kim, Department of Cell and Genetic Engineering, BK21 Project, Asan Medical Institute of Convergence Science and Technology, Asan Medical Center, University of Ulsan College of Medicine, Seoul 05505, Republic of Korea.
E-mail: yongsub1.kim@gmail.com

Published by Elsevier Inc. on behalf of The American Society of Gene and Cell Therapy.
This is an open access article under the CC BY license (http://creativecommons.org/licenses/by/4.0/).

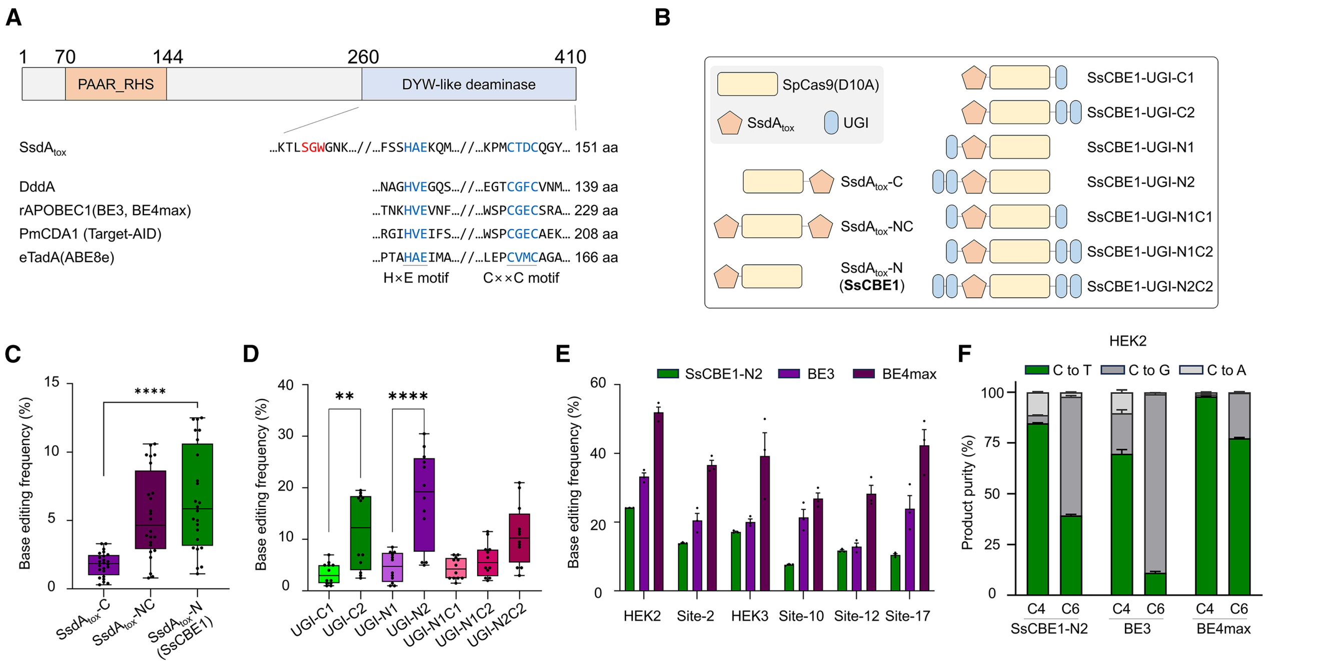

Figure 1. Use of the interbacterial toxin $SsdA_{tox}$ from the DYW-like deaminase clade in CRISPR cytosine base editor toolkits

(A) Domain analysis of full-length SsdA (410 aa), highlighting PAAR (proline-alanine-alanine-arginine), RHS (recombination hotspots), and the DYW-like deaminase toxin domain, referred to as $SSdA_{tox}$ (151 aa) in this study. A conserved Ser-Gly-Trp (SGW) motif was highlighted in red and His-x-Glu (HxE) and Cys-x-x-Cys (CxxC) motif was highlighted in blue. (B) Schematic overviews of constructing $SsdA_{tox}$-based cytosine base editors (CBEs). The constructs combine SpCas9 (D10A) nickase, $SsdA_{tox}$ domain, and UGI domains. (C) Base editing frequencies of each construct across 8 target sites in HEK293T/17-UNG knockout cells were depicted in a box and whisker plot. Dots represent the independent biological triplicate of each of 8 target sites. *****p* < 0.0001 by unpaired t test. (D) Base editing frequencies of each construct across 4 target sites in HEK293T/17 cells were depicted in a box and whisker plot. Dots represent the independent biological triplicate of each of 4 target sites. ***p* = 0.0011 and *****p* < 0.0001 by unpaired t test. (E) Base editing frequency of SsCBE1-N2, BE3, and BE4max compared in HEK293T/17 cells. (F) Product purity comparison of SsCBE1-N2, BE3, and BE4max at HEK2 C4 and C6 target sites. Data are represented as the mean of three independent biological replicate (*n* = 3) samples in bar graphs, with error bars representing the standard error of the mean (SEM).

CBE, BE4max, with reduced genome- and transcriptome-wide off-target effects. Finally, we demonstrate that SsCBE, when fused with TALE proteins, can be utilized for efficient cytosine base editing in mitochondrial DNA. Our findings contribute to expanding the base editing toolkit, paving the way for innovative applications in genome editing.

RESULTS

Evaluating a novel toxin-derived deaminase

We focused on a newly identified interbacterial cytidine deaminase toxin from the DYW-like subgroup encoded by *P. syringae* named $SsdA_{tox}$[15] (Figure 1A). Mougous and coworkers demonstrated that $SsdA_{tox}$ can induce C:G to T:A transitions in *Escherichia coli*, and showed *in vitro* deaminating activity toward ssDNA.[14] The $SsdA_{tox}$ comprises 151 amino acid (aa) residues, making it approximately 66% shorter than the rAPOBEC1 deaminase domain (229 aa) used in representative CBEs, BE3, and BE4max.[2,16] We initially confirmed the divergence of $SsdA_{tox}$ from other deaminases used in CRISPR-mediated base editing tools through phylogenetic tree analysis (Figure S1). To validate the deaminase activity of the $SsdA_{tox}$ domain, we purified it and conducted an *in vitro* deamination assay. Using an FAM-labeled ssDNA substrate, we confirmed that the $SsdA_{tox}$ domain has cytosine-to-uracil conversion activity (Figure S2). We then incubated $SsdA_{tox}$ protein with genomic DNA substrate from HEK293T/17 cells, dCas9 protein, and gRNA and analyzed the nucleotide sequences of target sites using targeted deep sequencing. The dCas9 and gRNA form an R-loop at genomic DNA target sites, exposing ssDNA substrates for $SsdA_{tox}$–induced cytosine-to-uracil conversion. We evaluated cytosine-to-thymine conversion frequencies at two target sites, RNF2 and HEK2, since uracil is read as thymine in sequencing. Targeted deep sequencing revealed that in the HEK2 and RNF2 sequences, despite that the activity of $SsdA_{tox}$ is restricted to the non-target strand and limiting conversion to 50%, cytosines were converted to thymine with a maximum frequency of 18.5% (Figures S3A and S3B). Targeted deep sequencing revealed that these conversion frequencies to thymine decreased to background levels after treatment with the uracil-specific excision reagent (USER) enzyme, confirming that $SsdA_{tox}$ protein indeed induced cytosine-to-uracil conversion, as the USER enzyme can recognize and eliminate uracil. These results underscore the potential of the $SsdA_{tox}$ domain for use in CBEs.

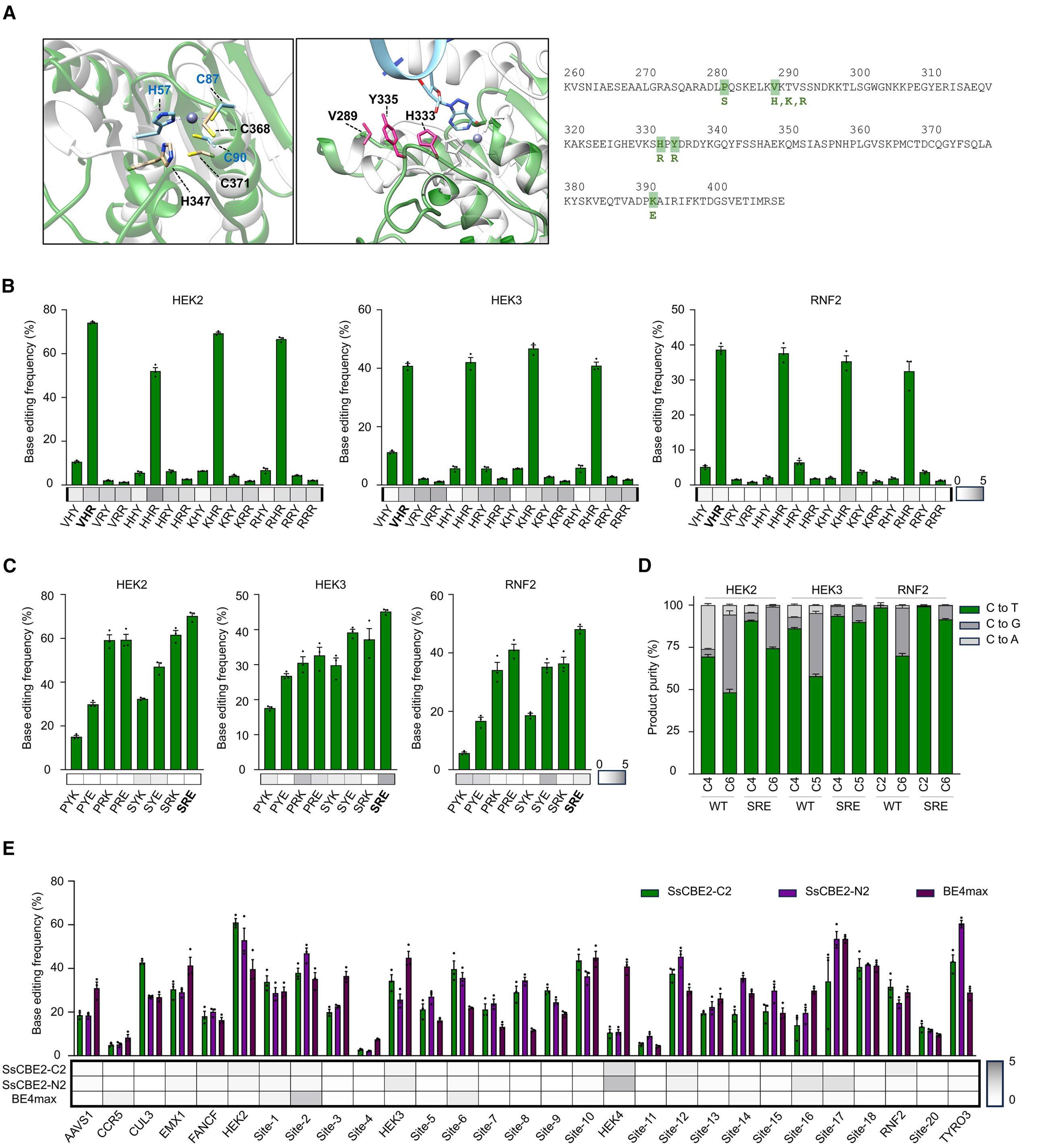

Figure 2. Enhancement of cytosine base editing activity in HEK293T/17 cells through rational engineering of the SsdA$_{tox}$ domain

(A) Structural alignment of TadA from ABE8e (gray, PDB: 6VPC) and SsdA$_{tox}$ (green, PDB: 7JTU). Left: catalytic active sites (blue for TadA, black for SsdA$_{tox}$). Center: three candidate residues for engineering (V289, H333, and Y335). Right: five candidate positions for engineering highlighted and designed amino acid residues listed below. (B and C) Base editing and indel frequencies of engineered variants. (B) Rational engineering at V289, H333, and Y335 positions; (C) and P282 and K392. Data are presented as

(legend continued on next page)

Development of a toxin-based novel CBE

To develop a novel CBE, we constructed several combinations of $SsdA_{tox}$, uracil-DNA glycosylase inhibitor (UGI), and spCas9-D10A nickase domains (Figure 1B). We evaluated the base editing frequencies of three constructs, $SsdA_{tox}$-C ($SsdA_{tox}$ fused to the C-terminal of spCas9-D10A nickase), $SsdA_{tox}$-N ($SsdA_{tox}$ fused to the N-terminal of spCas9-D10A nickase), and $SsdA_{tox}$-NC ($SsdA_{tox}$ fused to both the N- and C-terminals of spCas9-D10A nickase), across eight endogenous sites in *UNG* knockout HEK293T/17 cells (Figure 1C). We found that $SsdA_{tox}$-C exhibited relatively lower cytosine base editing activity compared to the other two constructs, with $SsdA_{tox}$-N reaching up to 12.4% cytosine base editing frequency at the HEK3 target site (Figure S4A). To further optimize, we added the UGI domain to either the N terminus or C terminus of the $SsdA_{tox}$-N domain (hereafter referred to as SsCBE1) and examined their base editing efficiency across eight endogenous sites in *UNG* knockout HEK293T/17 cells (Figure S4B). The addition of an extra UGI domain enhanced base editing frequency (UGI-C1 vs. UGI-C2 and UGI-N1 vs. UGI-N2), with the SsCBE1-UGI-N2 construct exhibiting the highest activity among all tested variants. Although the base editing frequencies of UGI variants were examined in *UNG* knockout backgrounds, we observed that UGI composition slightly affected base editing frequencies; SsCBE1 with N-terminal UGI domains showed higher base editing frequencies than those with C-terminal UGI domains. These results suggest that UGI composition might affect protein expression of the construct itself or other factors involved in the base editing mechanism, thereby altering base editing frequencies.

Subsequently, we compared the base editing efficiency and product purity of SsCBE1-UGI-N2 (hereafter referred to as SsCBE1-N2) against BE3 and BE4max in wild-type HEK293T/17 cells across six endogenous target sites (Figures 1D, 1E, and S4C). The SsCBE1-N2 exhibited base editing frequencies up to 24.2% at the HEK2 target site; however, the base editing frequencies of SsCBE1-N2 were lower than those of the representative CBE, BE3, and BE4max. The product purity of SsCBE1-N2 showed that it generally had high product purity, although there was room for improvement; at the cytosine position 6 of HEK2 target sequences, SsCBE-N2 had better product purity than BE3 but not than BE4max (Figures 1F and S4D). Therefore, we decided to engineer the $SsdA_{tox}$ domain to develop CBEs with improved performance.

Engineering and characterization of $SsdA_{tox}$ variants

We compared the structure of the $SsdA_{tox}$ protein (Protein Data Bank [PDB]: 7JTU) with that of ABE8e (PDB: 6VPC), which contains a similarly sized and well-characterized TadA deaminase (TadA8e), to predict their active sites and DNA-binding moieties[14,17] (Figure 2A). Given the lack of structural information regarding the $SsdA_{tox}$ domain and its DNA binding, we utilized the structure of TadA deaminase domain, the closest domain based on phylogenetic analysis, as a template for rational engineering of SsdA (Figure S1). Given the high sequence and structural similarity of the $SsdA_{tox}$ active site to that of TadA8e, we hypothesized that the DNA backbone interactions at the active site would also be analogous. Based on this assumption, we selected three amino acids (V289, H333, and Y335) located closest to the predicted DNA interaction site for potential enhancement of substrate binding. These residues were chosen because of their proximity to the DNA backbone, and we aimed to increase the likelihood of DNA interaction by introducing positively charged residues (histidine, lysine, and arginine) to enhance electrostatic interactions with the negatively charged DNA phosphate backbone.

To address the limitations of evaluating multiple variant combinations simultaneousl, we initially cloned 5 variants (V289H, V289K, V289R, H333R, and Y335R) and 10 combined variants of $SsdA_{tox}$ into the SsCBE1-UGI-C2 construct (hereafter referred to as SsCBE1-C2) and evaluated their base editing frequencies across three endogenous sites in HEK293T/17 cells (Figure 2B). Remarkably, the Y335R mutation increased cytosine base editing frequencies at the HEK2, HEK3, and RNF2 sites by 7.0-, 3.6-, and 7.5-fold, respectively, compared to wild-type $SsdA_{tox}$.

During subcloning of the $SsdA_{tox}$ variants, we observed that they exhibited toxicity in *E. coli* and that some C–to–T substitutions were occurring in the construct. We speculated that the $SsdA_{tox}$ constructs, which originally from a bacterial deaminase toxin, were weakly expressed in *E. coli* under the control of a mammalian promoter, leading to these issues. We then hypothesized that mutations induced in the construct from surviving *E. coli* cells were responsible for releasing their toxicity. Therefore, we decided to combine these mutations, P282S (<u>C</u>CT to <u>T</u>CT) and K392E (<u>A</u>AG to <u>G</u>AG), with the Y335R mutation.

Evaluation of 7 variants with combinations of P282S, K392E, and Y335R mutations showed that both P282S and K392E mutations enhanced base editing frequencies (Figure S5A). As expected, we confirmed that the P282S and K392E variants exhibited lower toxicity and increased expression levels compared to the wild-type $SsdA_{tox}$ (Figures S5B and S5C). The final engineered $SsdA_{tox}$ variant (hereafter referred to as SsCBE2-C2), featuring mutations P282S, Y335R, and K392E (named the SRE variant), exhibited an average 4.1-fold improvement in cytosine base editing efficiency and enhanced product purity compared to the wild-type in HEK293T/17 cells across three endogenous sites (Figures 2C, 2D, and S6A).

means, with error bars representing SEMs of three independent biological replicates (n = 3). Heatmaps below each bar plot show indel frequencies. (D) Comparison of product purity between wild-type and SRE variant across three target sites. (E) Base editing frequencies of SsCBE2-C2, SsCBE2-N2, and BE4max across 29 endogenous target sites in HEK293T/17 cells. Data are presented as means, with error bars representing SEMs of three independent biological replicates (n = 3). Indel frequencies are described below each bar plot.

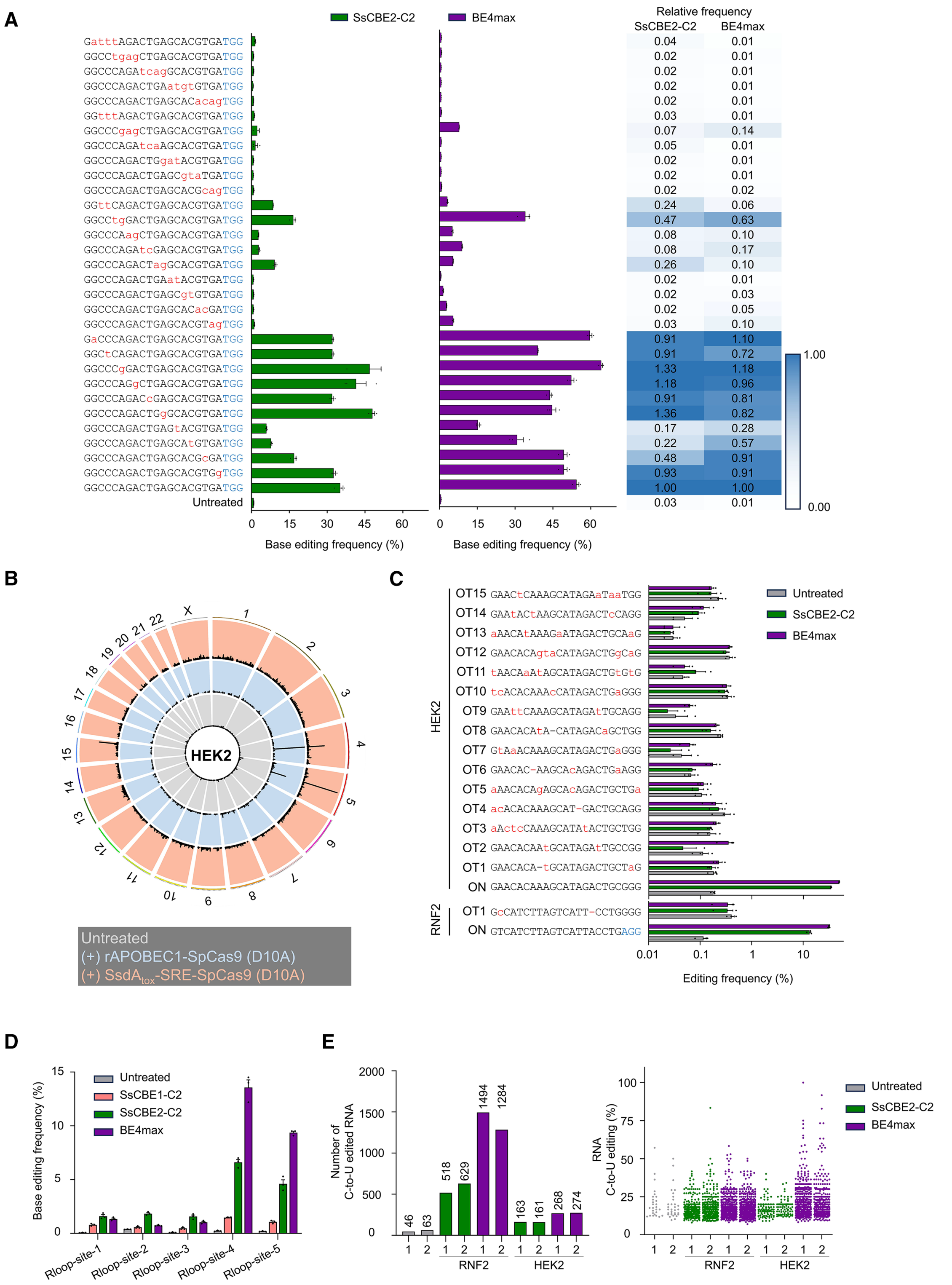

Figure 3. Identification of off-target effects of SsCBE2-C2

(A) Mismatch tolerance of SsCBE2-C2 and BE4max toward sgRNAs with 1–4 nt mismatches from the HEK3 site in HEK293T/17 cells. Protospacer adjacent motif (PAM) sequences are indicated in blue, and mismatched bases are indicated in red. Relative frequencies were calculated by dividing base editing frequencies obtained with

(legend continued on next page)

Given that the $SsdA_{tox}$ domain is derived from a toxin, we evaluated the cytotoxicity and the expression of several constructs in HEK293T/17 cells. Interestingly, protein engineering improved both the cytotoxicity and the expression levels of the $SsdA_{tox}$ domain; the SRE variants appeared to have a cell viability similar to that of BE4max (Figure S5B). We then incorporated the SRE variants into the SsCBE1-N2 construct, named SsCBE2-N2, and evaluated their base editing efficiency in HEK293T/17 cells across 29 endogenous target sites (Figure 2E). While wild-type $SsdA_{tox}$ demonstrated enhanced performance with an N-terminal UGI domain, SRE variants showed comparable performance with both N- and C-terminal UGI domains. Additionally, both SsCBE2-N2 and SsCBE2-C2 exhibited comparable base editing efficiency and indel frequencies to those of BE4max (Figure S6B). The base editing window of these variants appeared to be cytosine position 4–8, with slight editing observed at cytosine positions 3 and 9 of target sequences (Figure S6C). As is known, BE4max showed lower base editing frequency in a GC context[2]; however, SsCBE2 did not exhibit a context preference (Figure S6D). We also demonstrated that SsCBE2-C2 could induce high-frequency cytosine base editing in other cell lines, including K562, SKOV3, and HeLa cells (Figure S7). These findings indicate that rational engineering of the $SsdA_{tox}$ domain leads to significant improvements, yielding base editing frequencies comparable to those of BE4max.

gRNA-dependent off-target effects of SsCBE2

To assess the specificity of SsCBE2, we first investigated whether SsCBE2-C2 can tolerate mismatches in the spacer sequence of gRNAs. We transfected HEK293T/17 cells with plasmids encoding SsCBE2-C2 or BE4max, together with plasmids encoding the corresponding gRNA, each containing 0–4 mismatches in the spacer sequences. We then determined the substitution frequencies at two endogenous target sites (Figures 3A and S8A). Overall, SsCBE2-C2 generally exhibited less tolerance for mismatched targets than BE4max at the RNF2 site. For instance, the relative frequencies of SsCBE2-C2 and BE4max-induced substitutions at mismatched versus matched sites were 0.05 and 0.60, respectively, for a gRNA containing a single mismatch (at position 6, numbered 1–20 in the 5′ to 3′ direction) at the RNF2 site (Figure S8A). At the HEK2 site, SsCBE2-C2 exhibited less mismatch tolerance than BE4max for 4 out of 10 gRNAs containing a single mismatch (at positions 2, 12, 14, 16), but it showed similar or higher mismatch tolerance for the remaining gRNAs. Like BE4max, SsCBE-C2 showed low base editing activity for gRNAs containing two or three mismatches. These results suggest that SsCBE2-C2 enables precise genome editing.

To determine whether Digenome-seq could effectively evaluate the genome-wide target specificities of SsCBE2-C2, we incubated human genomic DNA from HEK293T/17 cells with ribonucleoproteins (RNPs) composed of purified SsCBE2-C2 protein and *in vitro*-transcribed gRNA, followed by treatment with the USER enzyme[18] (Figure S8B). Following the Digenome-seq protocol, which induces deamination *in vitro*, we employed constructs of $SsdA_{tox}$-SRE-spCas9 (D10A) and rAPOBEC1-SpCas9 (D10A) without the UGI domain. After whole-genome sequencing (WGS) the digested genomic DNA, we aligned the sequence reads to the human reference genome and used the Integrative Genomics Viewer to examine the alignment patterns at the on-target site.[19] The alignments indicated specific cleavage by SsCBE2-C2 and USER enzymes at the on-target site. To identify the off-target sites of SsCBE2-C2 in the human genome, we utilized a DNA cleavage score previously employed in our research (Figure 3B).[18,20,21] Based on the Digenome-seq method, we observed 2 and 16 cleavage sites for SsCBE2-C2 targeting RNF2 and HEK2, respectively (Figure S8C; Table S1). For instance, in Figure 3B, three major peaks can be observed. The peak on chromosome 5 corresponds to the on-target cleavage, while the peaks on chromosomes 4 and 15 are likely to represent potential off-target sites. To validate the off-target effects captured by Digenome-seq, we used targeted deep sequencing and determined SsCBE2-C2- and BE4max-induced substitution frequencies in HEK293T/17 cells. We examined 15 potential off-target sites and confirmed that none of these sites were validated by targeted deep sequencing with SsCBE2-C2 (Figure 3C). In contrast, BE4max induced two validated off-target events (HEK2-OT-II and HEK2-OT6) containing either two base pair mismatches or two base pair mismatches with one base pair RNA bulge, with observed frequencies of 0.35% and 0.18%, respectively. These findings underscore the high specificity of SsCBE2-C2.

gRNA-independent DNA and RNA off-target effects of SsCBE

To compare the gRNA-independent DNA deamination of SsCBE2-C2 and BE4max, we conducted an orthogonal R-loop assay using catalytically inactive *Staphylococcus aureus* Cas9 (dsaCas9) and saCas9 gRNA.[22] We assessed the gRNA-independent DNA deamination of SsCBE2-C2 and BE4max at artificially induced ssDNA sites by transfecting HEK293T/17 cells with plasmid DNA encoding either SsCBE2-C2 or BE4max, alongside spCas9 gRNA, dsaCas9, and saCas9 gRNA. Deamination frequencies in the R-loop formed by dsaCas9 were measured across 5 endogenous sites using targeted deep sequencing (Figure 3D). Notably, SsCBE2-C2 exhibited higher gRNA-independent off-target deamination at sites 1, 2, and 3 compared to BE4max but lower gRNA-independent off-target

mismatched sgRNAs by the mean base editing frequency of the matched sgRNA. Data are presented as means, with error bars representing SEMs of three independent biological replicates (*n* = 3). (B) Representative Circos plot illustrating genome-wide DNA cleavage scores obtained by Digenome-seq. Digenome-seq was performed with intact genomic DNA (gray), rAPOBEC-SpCas9 (D10A) plus hAAG and Endo VIII (blue), or with SsCBE2-SpCas9 (D10A) plus hAAG and Endo VIII (red). (C) Editing frequencies at off-target sites captured by Digenome-seq were measured by targeted deep sequencing in HEK293T/17 cells. PAM sequences are indicated in blue, and mismatched bases are indicated in red. Dashes represent RNA bulges. Data are presented as means, with error bars representing SEMs of three independent biological replicates (*n* = 3). (D) Measurement of Cas9-independent off-target deamination by dSaCas9-mediated orthogonal R-loop assay in HEK293T/17 cells. Data are presented as means, with error bars representing SEMs of three independent biological replicates (*n* = 3). (E) Cas9-independent RNA off-target deamination of SsCBE2-C2 and BE4max in HEK293T/17 cells. Transcriptome sequencing was used to determine the number of C-to-U edited nucleotides and the frequency of RNA C-to-U editing.

deamination at sites 5 and 6. These results indicate that the gRNA-independent DNA deamination of SsCBE2-C2 is comparable to that of BE4max.

Previous studies have shown that rAPOBEC1-based CBEs cause transcriptome-wide deamination, resulting in C-to-U conversion in a gRNA-independent manner.[23,24] To assess the gRNA-independent RNA off-target effects of SsCBE2-C2 and BE4max, HEK293T/17 cells were transfected with plasmids encoding SsCBE2-C2 or BE4-max along with corresponding gRNAs targeting RNF2 or HEK2. Total RNA was isolated 2 days post-transfection, and RNA sequencing (RNA-seq) was performed to assess transcriptome-wide RNA off-target editing; the Genome Analysis Toolkit (GATK) was used for RNA variant calling. Our analysis revealed that gRNA-independent C-to-U RNA editing induced by SsCBE2-C2 is lower than that induced by BE4max (Figures 3E and S9A). Additionally, the extent of A-to-G RNA editing was similar between BE4max-treated and untreated HEK293T/17 cells (Figure S9B). These findings suggest that the gRNA-independent RNA off-target C-to-U deamination of SsCBE2-C2 is lower than that of BE4max.

The versatility of SsCBE1-SRE

Given the need to miniaturize CRISPR systems for therapeutic applications, we explored the possibility of reducing the size of the $SsdA_{tox}$ domain through truncation. Through systematic N- and C-terminal truncations of the $SsdA_{tox}$ domain, we confirmed that its size could be further reduced by 5 aa without compromising base editing efficiency (Figure 4A). The Cas9 derived from *Campylobacter jejuni* (cjCas9) is one of the smallest Cas9 orthologs, making it a promising tool for *in vivo* therapy. We have previously developed a cjCas9-based CBE, cjCBEmax, which has an N-terminal rAPOBEC1 domain and a C-terminal tandem UGI domain of the cjCas9-D8A-L58Y/D900K nickase, cjCBEmax.[25] We replaced rAPOBEC1 with the SRE variant to generate cjSsCBE2 and evaluated its editing efficiency in HEK293T/17 cells across 4 endogenous sites. The cjSsCBE2 exhibited a 2.2-fold average improvement in cytosine base editing efficiency (Figures 4B and S10A). Subsequently, we produced adeno-associated virus (AAV) particles using cjSsCBE2-HPD or -ANGPT2, infected HEK293T/17 cells, and found that the base editing frequencies increased in a dose-dependent manner (Figures 4C and S10B).

Cas9 nuclease and BEs are used for RNP delivery, which is known to reduce off-target effects and cytotoxicity compared to plasmid DNA delivery.[21,26,27] To enhance the specificity of SsCBE2-C2, we employed RNP delivery by transfecting preassembled SsCBE2-C2 protein and *in vitro*-transcribed gRNA into HEK293T/17 cells. The results indicate that SsCBE2-C2 RNP delivery showed activity similar to that of plasmid DNA delivery (Figure 4D). We then generated engineered virus-like particles (eVLP) using SsCBE2-C2 to explore *in vivo* gene therapy potential.[28] After infecting HEK293T/17 cells with eVLPs containing SsCBE2-C2 protein and corresponding gRNA, we observed that eVLP-mediated base editing of SsCBE2-C2 exhibited comparable or higher activity than plasmid DNA delivery depending on the target sites.

To investigate the cytosine base editing capabilities of SsCBE2-C2 *in vivo*, we tried to induce a premature stop codon through a single C-to-T conversion at the tyrosinase (*Tyr*) gene (Tyr^{Q68*}) in mouse zygotes. As illustrated in Figure 4E, we utilized a gRNA known to be specifically designed to target the mouse *Tyr* gene and co-delivered SsCBE2-C2 mRNA along with this gRNA into mouse zygotes obtained from the C57BL/6NTac (B6N) mouse strain.[29] No acute toxicity was observed following the microinjection of SsCBE2-C2 mRNA into the B6 mouse zygotes (Figure 4F). We observed the edited allele in 5 out of 28 mice and embryos (17.9%), compared to a frequency of 18.0% (9 out of 50 embryos) when using the BE3, and successfully generated Tyr^{Q68*} mice using SsCBE2-C2 (Figures 4F–4H). These Tyr^{Q68*} mice exhibited the expected albino phenotype, consistent with the loss of functional tyrosinase activity. The developmental rate of the SsCBE2-C2 mRNA-injected mouse embryos was relatively lower than that of embryos edited with the BE3. However, given the sensitivity of B6 embryos, these findings suggest that SsCBE2-C2 is a suitable tool for gene editing in mouse fertilized eggs. This indicates that SsCBE2-C2 has potential for efficient base editing *in vivo*, offering an alternative to existing BEs with potentially enhanced specificity or efficiency for targeted gene modifications in mice.

Mitochondrial DNA base editing using SRE variants

Recent biochemical studies conducted *in vitro* have shown that the enzyme $SsdA_{tox}$ possesses cytosine deamination activity on ssDNA. Interestingly, at elevated concentrations, $SsdA_{tox}$ also exhibits the ability to deaminate cytosine in dsDNA.[14,30] To further explore the potential of SRE variant in mediating cytosine deamination within dsDNA genomes, we engineered fusions of SRE variant with TALE constructs, named TALE-SRE, akin to DdCBE systems, which incorporates UGI and a nuclear localization signal (NLS).[5] These TALE arrays were assembled using the high-throughput Golden Gate assembly method,[31] targeting three endogenous genomic sites (HEK3, HEK4, and TYRO3). Upon comparing with the DdCBE system, known for inducing high-frequency base editing at targeted loci, our constructs did not exhibit significant base editing activity at these genomic sites (Figure S11A). Subsequently, we constructed TALE arrays targeting mitochondrial DNA sites, including ND1, COX3, CYB, and ATP6, and fused these with the SRE variant, UGI, and a mitochondrial transport signal. Following transfection into HEK293T/17 cells, we quantified nucleotide conversion frequencies. Remarkably, the TALE-SRE-UGI constructs achieved C-to-T conversion rates of up to 4.3% at the targeted cytosine positions within 8 targeted mitochondrial DNA sites (Figures 5 and S11B). Contrary to the DdCBE system, which functioned as a dimer originally, our observations indicate that TALE-SRE can operate effectively as monomers at the target sites. While TALE-SRE exhibits lower efficiency compared to TALE-DdCBE, its monomeric nature and the potential for future optimization make it a promising candidate for further development. These findings underscore the versatility of the SRE variant, highlighting its capacity to target both ssDNA and dsDNA within the eukaryotic cellular environment.

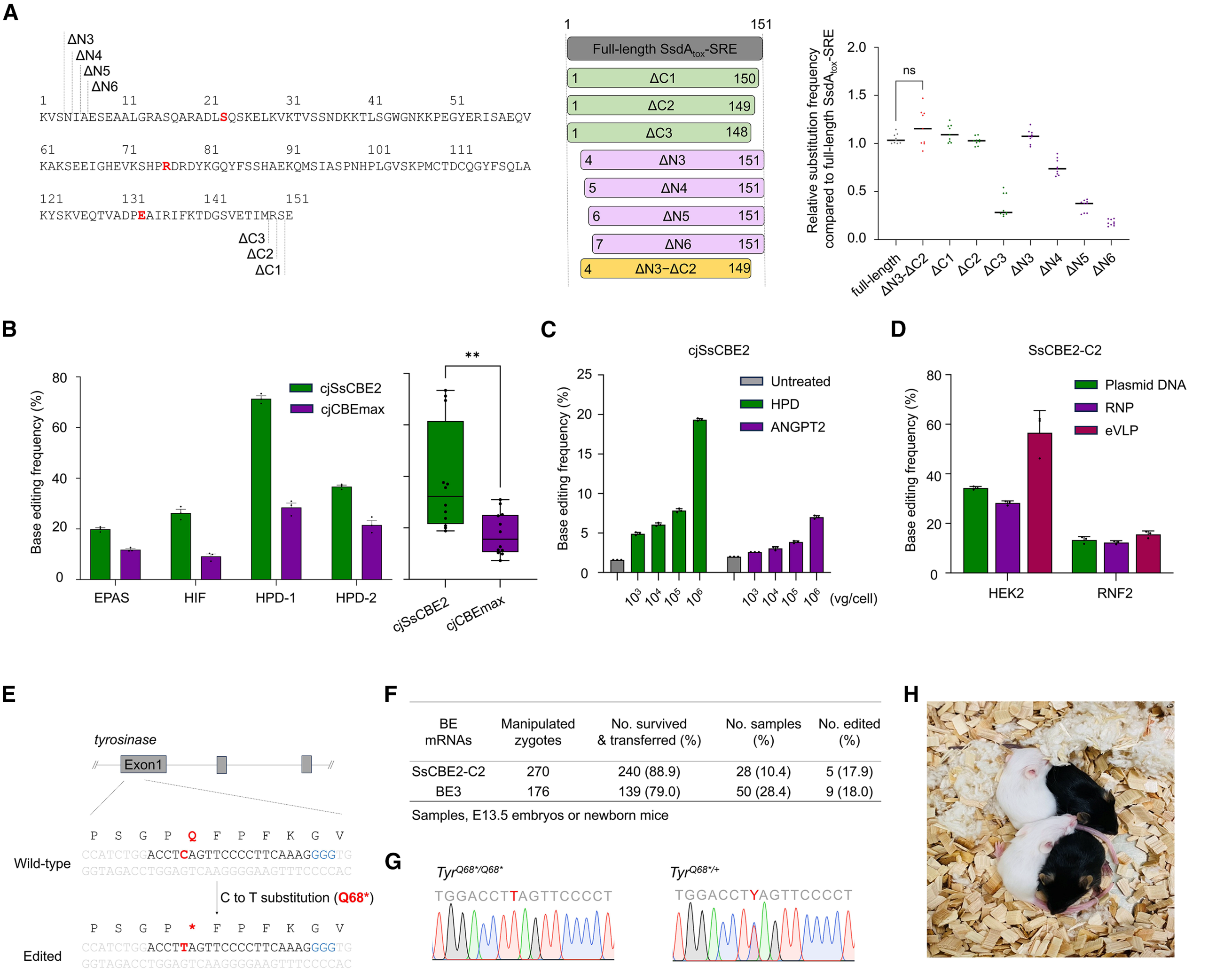

Figure 4. The versatility of the SsCBE2

(A) Either N- or C-terminally truncated SsdA$_{tox}$-SRE domains were cloned into SsCBE2-C2, and their base editing frequencies across three target sites in HEK293T/17 cells were measured by targeted deep sequencing. The aa sequences of the full-length SsdA$_{tox}$-SRE domains are presented at left (151 aa). SRE variants are highlighted in red, and the truncated sites are indicated. Relative frequencies were calculated by dividing base editing frequencies obtained with each variant by the mean base editing frequency obtained with full-length SsdA$_{tox}$-SRE in each target site. (B) Base editing frequency of cjSsCBE2 and cjCBEmax at 4 target sites in HEK293T/17 cells. Data represent means and error bars indicate SEMs of independent biological triplicates (n = 3). $^{**}p$ = 0.0040 by unpaired t test. (C) AAV particles produced with pAAV-cjSsCBE2-UGIx1-HPD or pAAV-cjSsCBE2-UGIx1-ANGPT2 were transduced at different vg/cells in HEK293T/17 cells, and base editing frequencies were measured by targeted deep sequencing. Data are plotted as means and error bars represent SEMs of independent biological triplicates (n = 3). (D) Comparison of SsCBE2-C2 base editing frequencies according to three delivery methods in HEK293T/17 cells; plasmids DNA, RNP, or eVLP. Data are presented as means, with error bars representing SEMs of three independent biological replicates (n = 3). (E) Schematic overviews of tyrosinase gene disruption by SsCBE2-mediate cytosine base editing. PAM sequences are highlighted in blue, and target cytosine is indicated in red. C-to-T conversion at target cytosine induces the nonsense mutation (Q68*). (F) Summarized results of microinjection of SsCBE2-C2 or BE3 mRNA in mouse one-cell embryos. (G and H) The nucleotide sequences of target sequences from pups were confirmed by Sanger sequencing. The C-to-T converted positions are highlighted in red.

DISCUSSION

Previously identified DYW deaminase family members are known to target RNA, but despite structural similarities to the family, SsdA$_{tox}$ from the DYW-like deaminase subgroup shows cytosine deaminase activity toward ssDNA substrates. Here, we report the development of a novel CRISPR BE utilizing the DYW-like deaminase. By fusing SsdA$_{tox}$ with nCas9, we demonstrated that the SsdA$_{tox}$-based BE can induce cytosine base editing in human cells. Furthermore, we enhanced the cytosine base editing activity and moderated the cytotoxicity through protein engineering of the SsdA$_{tox}$ domain, achieving comparable activity with the current representative CBE, BE4max. Using in-depth DNA and RNA off-target analysis, we

confirmed that the newly developed CBE, SsCBE2-C2, had comparable specificity compared to BE4max. Additionally, we showcased the versatility of the newly developed CBE by editing endogenous target sites using both AAV particles from a single cjSsCBE2 vector and DNA-free delivery methods, including RNP and eVLP delivery, highlighting the broad applicability of the $SsdA_{tox}$-based CBE.

As $SsdA_{tox}$ is derived from a bacterial toxin, we observed that the wild-type $SsdA_{tox}$ domain exhibited toxicity in *E. coli* during subcloning. Although the wild-type $SsdA_{tox}$ domain showed cytotoxicity in mammalian cells, we overcame this issue by incorporating a UGI domain and employing protein engineering (Figure S5A).

Recently, Gao and coworkers discovered a number of small and efficient deaminases using AlphaFold2, which were utilized for cytosine base editing[32]; however, the DYW-like deaminase we used, $SsdA_{tox}$, was not included in their findings. Upon experimenting with a truncated version of $SsdA_{tox}$, which is similar in size or slightly larger than other deaminases, we confirmed that its size could be further reduced by 5 aa without compromising base editing efficiency (Figure 4A).

Most deaminases developed for use in CBEs belong to the AID/APOBEC family, which share highly similar structures and functions[33] (Figure S1). Recently, structure-based protein clustering analyses have identified new deaminases that can be applied in base editing.[32,34,35] However, our discovery of $SsdA_{tox}$ did not arise from such analyses, indicating that while it shares some similarities with conventional deaminases, it also possesses distinct features. These differences suggest that $SsdA_{tox}$ offers unique advantages, setting it apart from previously identified deaminases and expanding the diversity of deaminase tools available for genome editing. This also implies that there may be additional, unexplored deaminase families that could be utilized for base editing.

While we were preparing this study, two independent groups also reported the use of $SsdA_{tox}$ for cytosine base editing in mammalian or plant systems.[36,37] Yin et al. resolved the crystal structure of $SsdA_{tox}$ bound to ssDNA and provided valuable mechanistic insights, while Zhang et al. introduced a G103S variant with demonstrated activity in plant systems and limited validation in mammalian cells. In contrast, our study presents a multi-site engineered variant with improved editing efficiency and reduced cytotoxicity, and it demonstrates its broad applicability across delivery methods and cellular compartments, including mitochondrial and *in vivo* systems. Collectively, these studies establish $SsdA_{tox}$ as a promising base editing scaffold, with our work representing one of the earliest and most comprehensive demonstrations of its potential. This also implies that there may be additional, unexplored deaminase families that could be utilized for base editing.

Given that the $SsdA_{tox}$ domain utilizes ssDNA as a template, its efficiency when bound to TALE was uncertain. Surprisingly, TALE-SRE demonstrated base editing efficiency in mitochondrial DNA but not in the nuclear genome. Previous studies have shown that although $SsdA_{tox}$ primarily uses ssDNA as a template, it exhibits very weak activity with dsDNA substrates. We hypothesize that this phenomenon might explain why TALE-SRE is efficient in mitochondrial DNA. However, we believe that further research is necessary to fully understand the mechanism underlying TALE-SRE function in mitochondrial DNA.

Unexpectedly, the TALE-SRE, which may target dsDNA as a substrate, showed cytosine base editing activity, but we cannot find any genome-wide off-target effect through *in vitro* Digenome-seq. This finding is noteworthy, since previous studies demonstrated that the $SsdA_{tox}$ domain targets dsDNA with substantially lower efficiency than ssDNA. Furthermore, the development of a small CBE opens up new possibilities for base editing applications, not only in the nucleus but also in cellular organelles.

Conclusions

In conclusion, this study presents a CRISPR-based CBE (SsCBE) utilizing $SsdA_{tox}$, a DYW-like deaminase derived from *P. syringae*. Through rational engineering, SsCBE demonstrated improved base editing efficiency and reduced cytotoxicity, offering versatility across various delivery methods. Its successful application in both nuclear and mitochondrial DNA editing highlights the potential of SsCBE as a powerful tool for genome engineering and therapeutic strategies. This advancement broadens the CRISPR toolkit, paving the way for further innovations in precision genetic modifications.

MATERIALS AND METHODS

Plasmid construction

The human codon-optimized $SsdA_{tox}$ domain was synthetized (Integrated DNA Technologies) and cloned into either the N terminus or C terminus of a modified pCMV_BE4max vector (Addgene, catalog no. 112093), with the UGI domain deleted. To construct $SsdA_{tox}$-UGI variants, one or two copies of the UGI domain were amplified by Phusion High-Fidelity DNA Polymerase (Thermo Fisher Scientific) and cloned into designated positions. For constructing rationally engineered SsCBE-UGI-C2 variants, primers containing mismatches with wild-type sequences were used to introduce mutations into the $SsdA_{tox}$ sequences. The amplicons were then cloned into the wild-type SsCBE-UGI-C2 vector using Gibson Assembly Master Mix (New England Biolabs). The cjSsCBE2 was constructed by exchanging the APOBEC1 domain of cjCBEmax with the $SsdA_{tox}$-SRE domain, and the pAAV-cjABE8e-gRNA-ANGPT2-HPD-2 was modified to construct the single AAV vector encoding cjSsCBE2.[25] The gRNAs were constructed using the pRG2Z vector (Addgene, catalog no. 104174) and pU6-Cj-sgRNA (single-guide RNA; Addgene, catalog no. 89753). The target sequences used in this study are listed in Table S2.

Cell culture and transfection

HEK293T/17 and HeLa were maintained in DMEM medium with 10% fetal bovine serum (FBS) and 1% penicillin-streptomycin. K562 and SKOV3 were maintained in RPMI and McCoy's 5A medium,

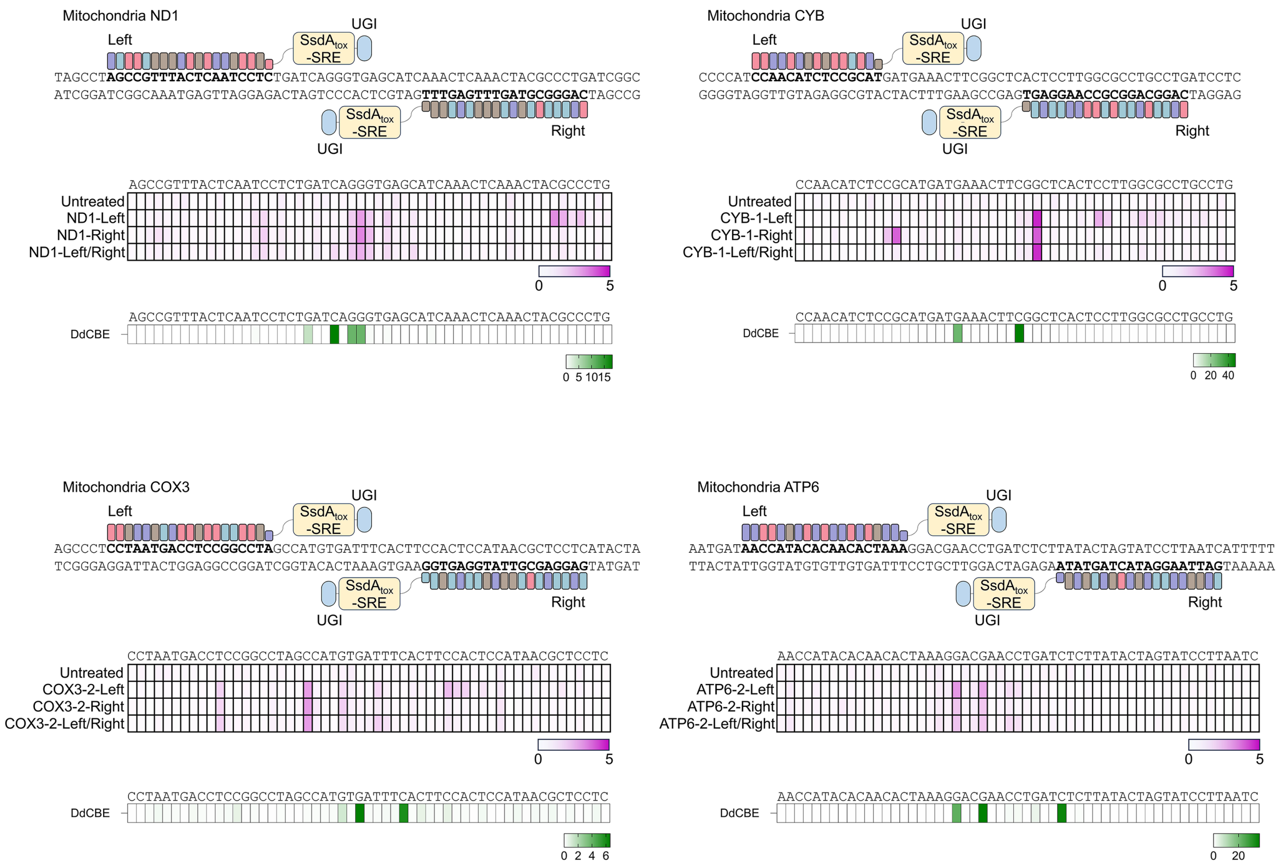

Figure 5. Mitochondrial genome editing using TALE-SRE in HEK293T/17 cells

Architectures of TALE-SRE targeting the ND1, CYB, COX3, and ATP6 sites are described. Monomer (left-TALE-SRE or right-TALE-SRE) and dimer (left-TALE-SRE and right-TALE-SRE) forms of TALE-SRE were transfected in HEK293T/17 cells at three independent biological replicates, and base editing frequencies were measured by targeted deep sequencing and described in heatmaps. Dimeric DdCBE constructs served as control.

respectively, with 10% FBS and 1% penicillin-streptomycin. All the mammalian cells were incubated at 37°C in a 0.05% CO_2 atmosphere and routinely tested for Mycoplasma contamination using MycoStrip (InvivoGen). The cells were seeded onto 48-well plates (Corning) 1 day before transfection, and transfection was conducted at 50%–60% cell confluency using Lipofectamine 2000 (Thermo Fisher Scientific) unless otherwise stated. Briefly, a total of 500 ng plasmid DNA (250 ng of gRNA and 250 ng of BEs or each 250 ng of TALE-SREs) were mixed with 1.5 μL Lipofectamine 2000. For K562 cells, 2×10^5 cells were electroporated with 250 ng gRNA plasmids and 750 ng CBE using the SF Cell Line Nucleofector X Kit (Lonza) via the 4D-Nucleofector system. Genomic DNA was extracted 96 h post-transfection using homemade cell lysis buffer (10 mM Tris-HCl pH 8.5 and 0.05% SDS) or DNeasy Blood & Tissue Kits (Qiagen) to evaluate editing frequencies. For evaluating cell viability, cells were subjected to a luminescent assay using CellTiter-Glo 2.0 (Promega) 72 h post-transfection according to the manufacturer's protocol. To compare the expression levels of each $SsdA_{tox}$ variants, the P2A-mCherry fused variants were transfected and subjected to FACS (BD FACSCanto) analysis 48 h post-transfection.

Targeted deep sequencing

Genomic DNA containing either the on-target or off-target sites was amplified with KAPA HiFi HotStart DNA polymerase (Roche) or SUN-PCR Blent (SUN GENETICS) according to the manufacturer's instructions. The amplified products, including Illumina TruSeq HT dual index adapter sequences, were subjected to 150-bp paired-end sequencing using the Illumina iSeq 100 platform. MAUND, an analysis tool accessible at https://github.com/ibs–cge/maund, was used to determine the base editing efficiencies. The primer sequences used in this study are listed in Tables S2 and S3.

AAV particle production and transduction

HEK293T/17 cells were seeded onto a 150-mm culture dish 1 day before transfection, and pAAV-cjSsCBE2-ANGPT2 or pAAV-cjSsCBE2-HPD was transfected with pAAV-DJ and helper plasmids. The transfection was conducted at 70% cell confluency and with plasmids at the molar ratio of 1:1:1. AAV particles were collected and concentrated 72 h post-transfection using the AAVpro Purification Kit Midi (Takara) according to the manufacturer's protocol. We seeded 1×10^4 HEK293T/17 cells in 96-well plates and AAV

particles at different vector genomes (vg)/cell were transduced. Cells were collected 96h after transduction to measure cytosine base editing frequency. The vg/cell was determined by real-time PCR using the AAVpro Titration Kit (Takara) according to the manufacturer's protocol. HEK293T/17 cells used in the experiments regarding AAV production and transduction were maintained in DMEM with 2% FBS.

Protein purification

The plasmid encoding the pABE8e-protein (Addgene plasmid no. 161788) was used to construct the plasmid encoding the His_8-SsCBE2-C2, by the Gibson assembly method. The transformed Rosetta cells (EMD Millipore) were grown overnight at 37°C in Luria-Bertani (LB) broth supplemented with 100 μg/mL kanamycin and 34 μg/mL chloramphenicol after transformation with the His_8-SsCBE2-C2 plasmid DNA. Subsequently, 10 mL of overnight cultures of Rosetta cells transformed with His_8-SsCBE2-C2 plasmid DNA were inoculated into 400 mL of LB broth supplemented with 100 μg/mL kanamycin and 34 μg/mL chloramphenicol at 30°C until the optical density at 600 nm reached 0.5–0.6. The cells were cooled to 18°C for 1 h, followed by induction of His_8-SsCBE2-C2 protein with 0.8% rhamnose, and subsequent culture for another 18 h. For protein purification, cells were harvested by centrifugation at 5,000 × *g* for 10 min at 4°C and lysed via sonication in 5 mL lysis buffer (50 mM NaH_2PO_4, 300 mM NaCl, 1 mM DTT, and 10 mM imidazole, pH 8.0) supplemented with lysozyme (Sigma) and protease inhibitor (Roche complete, EDTA-free). The soluble lysate obtained after centrifugation at 13,000 rpm for 30 min at 4°C was incubated with Ni-NTA agarose resin (Qiagen) for 1 h at 4°C. The mixture of lysate and Ni-NTA was applied onto a column and washed with a buffer containing 50 mM NaH_2PO_4, 300 mM NaCl, and 20 mM imidazole at pH 8.0. The SsCBE2-C2 protein was subsequently extracted by utilizing the elution buffer (50 mM NaH_2PO_4, 300 mM NaCl, and 250 mM imidazole, pH 8.0). To improve the purity of the SsCBE2-C2 protein, we subjected fractions containing the protein to additional purification steps. These fractions were combined with heparin beads (Cytiva) in a solution composed of 20 mM Tris-HCl (pH 8.0 at 25°C), 150 mM NaCl, 5% (v/v) glycerol, and 1% (v/v) Triton X-100. We eluted proteins by a linear gradient of NaCl concentration from 600 mM to 2 M in a buffer containing 20 mM Tris-HCl (pH 8.0 at 25°C), 0.1 mM DTT, and 5% (v/v) glycerol. The fractions that contained the SsCBE2-C2 protein were collected and concentrated through an Amicon Ultra centrifugal filter (Millipore). Following that, a buffer exchange with storage buffer (20 mM HEPES-KOH, pH 7.5, 150 mM KCl, 1 mM DTT, and 20% glycerol) was performed. The concentrated protein underwent further processing with centrifugal filter units (Millipore). The purified SsCBE2-C2 protein was then stored at −80°C.

Digenome-seq

The genomic DNA was extracted using the DNeasy Tissue Kit (Qiagen) following the manufacturer's protocols. To induce *in vitro* deamination, the SsCBE2-C2 protein (100 nM) was pre-incubated with gRNA (300 nM) for 10 min at room temperature. Subsequently, the preassembled complex was mixed with genomic DNA (10 μg) in a reaction buffer (100 mM NaCl, 50 mM Tris-HCl, 10 mM $MgCl_2$, and 100 μg/mL BSA) to a final reaction volume of 1,000 μL. The reaction mixture was incubated at 37°C for 8 h. After deamination, the genomic DNA was purified using the DNeasy Tissue Kit (Qiagen), and RNase A (50 μg/mL) was added for the elimination of gRNA. A second incubation step with USER enzyme (6 U) was performed on purified genomic DNA (2 μg), and the reaction volume was 100 μL, incubated at 37°C for 3 h. This was followed by another purification round using the DNeasy Blood & Tissue Kit (Qiagen). After digestion with SsCBE2-C2 and USER enzymes, the genomic DNA was subjected to WGS at 30–40× depth using an Illumina HiSeq X Ten sequencer at Macrogen. The genome sequence was mapped using the Isaac aligner, and the DNA cleavage sites were identified using the Digenome program, available at https://github.com/chizksh/digenome−toolkit2.

Orthogonal R-loop assay

dsaCas9 (Addgene plasmid no. 138162) and their gRNAs were used in an orthogonal R-loop assay.[22] A total of 500 ng plasmids (each 100 ng plasmids of dsaCas9, saCas9-gRNAs, SsCBE or BE4max, gRNAs, and p3s-EFS-puromycinR) were transfected in HEK293T/17 cells and puromycin was treated 24 h post-transfection at 1 μg/mL to select transfected cells. Genomic DNA was extracted 96 h after transfection, and editing frequencies were measured by targeted deep sequencing.

Transcriptome sequencing

Total RNA was extracted 48 h post-transfection using the RNeasy Mini Kit (Qiagen) according to the manufacturer's instructions. RNA libraries were then generated with the TruSeq Stranded Total RNA Library Prep Gold Kit (Illumina). Evaluation of RNA library quality was performed using the Agilent 2200 TapeStation with a D1000 ScreenTape system. Total RNA-seq was conducted at Macrogen using a NovaSeq 6000 Sequencer (Illumina) with paired-end sequencing (2 × 100 bp).

RNA variant calling

To analyze RNA sequencing data generated by next-generation sequencing (NGS), we used a previously validated RNA variant calling pipeline designed for the analysis of off-target RNA base editing.[23,38] The NGS data were aligned to the hg38 (release version 105) human reference genome using the STAR aligner (version 2.7.10a). BAM files were then processed for RNA variant calling using MarkDuplicates, BaseRecalibrator, ApplyBQSR, and HaplotypeCaller from the GATK package (version 4.2.4.1). We filtered RNA variant loci by comparing them with control samples. In the experimental set of replicate 1, the untreated replicate 2 served as the control, while in the experimental sets of replicate 2, the untreated replicate 1 served as the control. RNA variant loci with a variant count of at least 2 and a read depth of at least 10 were retained. We excluded RNA variant loci that were already present in the control sample or that were considered indeterminate due to low sequencing depth in the control sample. C-to-T editing was

quantified as RNA variant loci, with C-to-T editing on the positive strand or G-to-A editing on the negative strand among total RNA editing. Similarly, A-to-G editing was quantified as RNA variant loci, with A-to-G editing on the positive strand or T-to-C editing on the negative strand among total RNA editing.

SsCBE2-C2 eVLP production and purification

The plasmid containing pCMV–MMLVgag-3xNES-ABE8e was a gift from David Liu (Addgene plasmid no. 181751). Construction of the plasmid encoding pCMV-MMLVgag-3xNES-SsCBE2-C2 was accomplished through Gibson assembly. SsCBE2-C2-eVLPs were generated by transfecting Gesicle Producer 293T cells. Gesicle cells were seeded in a 150-mm cell culture dish at a density of 5×10^6 cells per dish. After 20–24 h, the cells were transfected using polyethylenimine (Sigma) according to the manufacturer's protocols. To produce SsCBE2-C2-eVLP, a mixture of plasmids expressing VSV-G (400 ng), MMLVgag-pro-pol (3,375 ng), MMLVgag-3xNES-SsCBE2-C2-eVLPs (1,125 ng), and a gRNA (4,400 ng) were co-transfected per 150-mm cell culture dish. At 40–48 h post-transfection, the supernatant from the transfected cells was harvested and subjected to centrifugation for 5 min at $500 \times g$ to eliminate cell debris. The resulting supernatant was then filtered through a 0.45-μm polyvinylidene fluoride filter. For concentration, the filtered supernatant underwent a 100-fold concentration step using PEG-it Virus Precipitation Solution (System Biosciences) according to the manufacturer's protocols.

Construction of TALE-SsCBE

Target-specific TALE-SsCBEs were assembled by one-step Golden Gate cloning with TALE RVD modules.[31] Empty expression vectors were first constructed according to the previous study by modifying Transcription Activator-Like Effector Nuclease expression vectors.[5] Briefly, fokI domains were exchanged by the $SsdA_{tox}$-SRE domain to construct TALE-SsCBE expression vectors. For nucleus genomes targeting TALE-SsCBEs, two copies of UGI were cloned between the NLS and N-terminal domains of TALE. For mitochondrial genomes targeting TALE-SsCBE, the mitochondrial targeting sequence was cloned instead of NLS, and one copy each of UGI and NES signal was added in the C terminus of the expression vectors. All the assembled TALE-SsCBEs were subjected to Sanger sequencing to confirm their sequences. The target sequences used in this study are listed in Table S3.

Generation of *Tyr*^{Q68*} mutant mice

All animal experiments were conducted according to the Korean Ministry of Food and Drug Safety guidelines, and animal protocols were reviewed and approved by the Institutional Animal Care and Use Committee of Asan Institute for Life Sciences (permit no. 2020-14-347). All mice were maintained in the specific pathogen-free facility of the Laboratory of Animal Research in the Asan Medical Center.

To construct the *in vitro* transcription template for the gRNA, the following pair of oligomers was annealed and cloned into pUC57-sgRNA vector (Addgene plasmid no. 51132): 5′-TAGGACCT CAGTTCCCCTTCAAAG-3′ and 5′-AAACCTTTGAAGGGGAAC TGAGGT-3′. The constructs encoding BE3 or SsCBE2-C2 were linearized using the PmeI restriction enzyme (New England Biolabs). mRNAs of BEs and gRNA were synthesized *in vitro* from linearized DNA templates using the mMESSAGE mMACHINE T7 Ultra kit (Ambion) and the MEGAshortscript T7 kit (Ambion), respectively, according to the manufacturer's protocol.

C57BL/6N (DBL) and ICR (OrientBio) mouse strains were used as embryo donors and foster mothers, respectively. Micromanipulation of fertilized eggs collected from female mice of C57BL/6N (B6N) strain and subsequent processes required for the mouse model establishment were performed as previously described.[39] The mixture of BE3 or SsCBE2-C2 mRNA (250 ng/μL) and gRNA (50 ng/μL) was diluted with RNase-free injection buffer (0.25 mM EDTA, 10 mM Tris, pH 7.4) and microinjected into male pronuclei using a TransferMan NK2 micromanipulator and a FemtoJet microinjector (Eppendorf). The manipulated embryos were transferred into the oviducts of pseudopregnant foster mothers. Genomic DNA samples were isolated from tail biopsies of newborn mice, and embryonic day 13.5 mouse embryos were analyzed by targeted deep sequencing.

Statistical analysis

All results are expressed as the mean ± SEM unless indicated otherwise. The statistical analysis was performed in GraphPad Prism 9.1.1. p values were derived using Student's two-tailed t test.

DATA AVAILABILITY

The high-throughput sequencing data from this study have been deposited in the NCBI Sequence Read Archive database under the accession code PRJNA1076329 and are publicly available as of the date of publication.

ACKNOWLEDGMENTS

This research was supported and funded by the National Research Foundation of Korea (grant nos. 2021R1C1C2013270 to J.K.; RS-2023-00210965 to K.L.; 2022M3A9E4082652 to Y.H.S.; 2020R1A2C2101714 to D.K.; and RS-2023-00260462, RS-2025-02218274, and RS-2024-00459998 to Y.K.), the Korea Health Technology R&D Project, Ministry of Health and Welfare, Republic of Korea (grant nos. HI21C1314 and HR22C1363 to D. K.), the Korea Institute of Science and Technology Institutional Program (grant no. 2E32161 to K.L.), and the Asan Institute for Life Sciences (Seoul, Republic of Korea) (grant nos. 2023IP0105 and 2024IP0075).

AUTHOR CONTRIBUTIONS

Y.K. supervised the research. J.K., D.K., and Y.K. conceived the research. J.K., S.P., Y.H. S., D.K., and Y.K. designed the study. J.K., S.P., D.K., and Y.K. performed and analyzed the main experiments. J.K., S.P., M.Y.J., K.L., A.-H.J., M.L., C.S., C.L., S.P., J.A., and J.J. performed the experiments. J.K., S.P., Y.H.S., D.K., and Y.K. wrote the manuscript.

DECLARATION OF INTERESTS

J.K., G.J., D.K., and Y.K. have filed patent applications related to this work.

SUPPLEMENTAL INFORMATION

Supplemental information can be found online at https://doi.org/10.1016/j.ymthe.2025.08.007.

REFERENCES

1. Porto, E.M., Komor, A.C., Slaymaker, I.M., and Yeo, G.W. (2020). Base editing: advances and therapeutic opportunities. Nat. Rev. Drug Discov. *19*, 839–859.
2. Komor, A.C., Kim, Y.B., Packer, M.S., Zuris, J.A., and Liu, D.R. (2016). Programmable editing of a target base in genomic DNA without double-stranded DNA cleavage. Nature *533*, 420–424.
3. Nishida, K., Arazoe, T., Yachie, N., Banno, S., Kakimoto, M., Tabata, M., Mochizuki, M., Miyabe, A., Araki, M., Hara, K.Y., et al. (2016). Targeted nucleotide editing using hybrid prokaryotic and vertebrate adaptive immune systems. Science *353*, aaf8729.
4. Gaudelli, N.M., Komor, A.C., Rees, H.A., Packer, M.S., Badran, A.H., Bryson, D.I., and Liu, D.R. (2017). Programmable base editing of A*T to G*C in genomic DNA without DNA cleavage. Nature *551*, 464–471.
5. Mok, B.Y., de Moraes, M.H., Zeng, J., Bosch, D.E., Kotrys, A.V., Raguram, A., Hsu, F., Radey, M.C., Peterson, S.B., Mootha, V.K., et al. (2020). A bacterial cytidine deaminase toxin enables CRISPR-free mitochondrial base editing. Nature *583*, 631–637.
6. Cho, S.I., Lee, S., Mok, Y.G., Lim, K., Lee, J., Lee, J.M., Chung, E., and Kim, J.S. (2022). Targeted A-to-G base editing in human mitochondrial DNA with programmable deaminases. Cell *185*, 1764–1776.e12.
7. Lim, K., Cho, S.I., and Kim, J.S. (2022). Nuclear and mitochondrial DNA editing in human cells with zinc finger deaminases. Nat. Commun. *13*, 366.
8. Willis, J.C.W., Silva-Pinheiro, P., Widdup, L., Minczuk, M., and Liu, D.R. (2022). Compact zinc finger base editors that edit mitochondrial or nuclear DNA in vitro and in vivo. Nat. Commun. *13*, 7204.
9. Budzko, L., Hoffa-Sobiech, K., Jackowiak, P., and Figlerowicz, M. (2023). Engineered deaminases as a key component of DNA and RNA editing tools. Mol. Ther. Nucleic Acids *34*, 102062.
10. Kim, J.S., and Chen, J. (2024). Base editing of organellar DNA with programmable deaminases. Nat. Rev. Mol. Cell Biol. *25*, 34–45.
11. Lam, D.K., Feliciano, P.R., Arif, A., Bohnuud, T., Fernandez, T.P., Gehrke, J.M., Grayson, P., Lee, K.D., Ortega, M.A., Sawyer, C., et al. (2023). Improved cytosine base editors generated from TadA variants. Nat. Biotechnol. *41*, 686–697.
12. Neugebauer, M.E., Hsu, A., Arbab, M., Krasnow, N.A., McElroy, A.N., Pandey, S., Doman, J.L., Huang, T.P., Raguram, A., Banskota, S., et al. (2023). Evolution of an adenine base editor into a small, efficient cytosine base editor with low off-target activity. Nat. Biotechnol. *41*, 673–685.
13. Chen, L., Zhu, B., Ru, G., Meng, H., Yan, Y., Hong, M., Zhang, D., Luan, C., Zhang, S., Wu, H., et al. (2023). Re-engineering the adenine deaminase TadA-8e for efficient and specific CRISPR-based cytosine base editing. Nat. Biotechnol. *41*, 663–672.
14. de Moraes, M.H., Hsu, F., Huang, D., Bosch, D.E., Zeng, J., Radey, M.C., Simon, N., Ledvina, H.E., Frick, J.P., Wiggins, P.A., et al. (2021). An interbacterial DNA deaminase toxin directly mutagenizes surviving target populations. Elife *10*, e62967.
15. Iyer, L.M., Zhang, D., Rogozin, I.B., and Aravind, L. (2011). Evolution of the deaminase fold and multiple origins of eukaryotic editing and mutagenic nucleic acid deaminases from bacterial toxin systems. Nucleic Acids Res. *39*, 9473–9497.
16. Koblan, L.W., Doman, J.L., Wilson, C., Levy, J.M., Tay, T., Newby, G.A., Maianti, J. P., Raguram, A., and Liu, D.R. (2018). Improving cytidine and adenine base editors by expression optimization and ancestral reconstruction. Nat. Biotechnol. *36*, 843–846.
17. Lapinaite, A., Knott, G.J., Palumbo, C.M., Lin-Shiao, E., Richter, M.F., Zhao, K.T., Beal, P.A., Liu, D.R., and Doudna, J.A. (2020). DNA capture by a CRISPR-Cas9-guided adenine base editor. Science *369*, 566–571.
18. Kim, D., Lim, K., Kim, S.T., Yoon, S.H., Kim, K., Ryu, S.M., and Kim, J.S. (2017). Genome-wide target specificities of CRISPR RNA-guided programmable deaminases. Nat. Biotechnol. *35*, 475–480.
19. Robinson, J.T., Thorvaldsdottir, H., Turner, D., and Mesirov, J.P. (2023). igv.js: an embeddable JavaScript implementation of the Integrative Genomics Viewer (IGV). Bioinformatics *39*, btac830.
20. Kim, D., Bae, S., Park, J., Kim, E., Kim, S., Yu, H.R., Hwang, J., Kim, J.I., and Kim, J.S. (2015). Digenome-seq: genome-wide profiling of CRISPR-Cas9 off-target effects in human cells. Nat. Methods *12*, 237–243.
21. Kim, D., Kim, D.E., Lee, G., Cho, S.I., and Kim, J.S. (2019). Genome-wide target specificity of CRISPR RNA-guided adenine base editors. Nat. Biotechnol. *37*, 430–435.
22. Doman, J.L., Raguram, A., Newby, G.A., and Liu, D.R. (2020). Evaluation and minimization of Cas9-independent off-target DNA editing by cytosine base editors. Nat. Biotechnol. *38*, 620–628.
23. Grunewald, J., Zhou, R., Garcia, S.P., Iyer, S., Lareau, C.A., Aryee, M.J., and Joung, J. K. (2019). Transcriptome-wide off-target RNA editing induced by CRISPR-guided DNA base editors. Nature *569*, 433–437.
24. Jin, S., Zong, Y., Gao, Q., Zhu, Z., Wang, Y., Qin, P., Liang, C., Wang, D., Qiu, J.L., Zhang, F., and Gao, C. (2019). Cytosine, but not adenine, base editors induce genome-wide off-target mutations in rice. Science *364*, 292–295.
25. Kweon, J., Jang, A.H., Kwon, E., Kim, U., Shin, H.R., See, J., Jang, G., Lee, C., Koo, T., Kim, S., and Kim, Y. (2023). Targeted dual base editing with Campylobacter jejuni Cas9 by single AAV-mediated delivery. Exp. Mol. Med. *55*, 377–384.
26. Rees, H.A., Komor, A.C., Yeh, W.H., Caetano-Lopes, J., Warman, M., Edge, A.S.B., and Liu, D.R. (2017). Improving the DNA specificity and applicability of base editing through protein engineering and protein delivery. Nat. Commun. *8*, 15790.
27. Kim, S., Kim, D., Cho, S.W., Kim, J., and Kim, J.S. (2014). Highly efficient RNA-guided genome editing in human cells via delivery of purified Cas9 ribonucleoproteins. Genome Res. *24*, 1012–1019.
28. Banskota, S., Raguram, A., Suh, S., Du, S.W., Davis, J.R., Choi, E.H., Wang, X., Nielsen, S.C., Newby, G.A., Randolph, P.B., et al. (2022). Engineered virus-like particles for efficient in vivo delivery of therapeutic proteins. Cell *185*, 250–265.e16.
29. Kim, K., Ryu, S.M., Kim, S.T., Baek, G., Kim, D., Lim, K., Chung, E., Kim, S., and Kim, J.S. (2017). Highly efficient RNA-guided base editing in mouse embryos. Nat. Biotechnol. *35*, 435–437.
30. Vaisvila, R., Johnson, S.R., Yan, B., Dai, N., Bourkia, B.M., Corrêa, I.R., Jr., Yigit, E., and Sun, Z. (2023). Discovery of novel DNA cytosine deaminase activities enables a nondestructive single-enzyme methylation sequencing method for base resolution high-coverage methylome mapping of cell-free and ultra-low input DNA. Preprint at bioRxiv. https://doi.org/10.1101/2023.06.29.547047.
31. Kim, Y., Kweon, J., Kim, A., Chon, J.K., Yoo, J.Y., Kim, H.J., Kim, S., Lee, C., Jeong, E., Chung, E., et al. (2013). A library of TAL effector nucleases spanning the human genome. Nat. Biotechnol. *31*, 251–258.
32. Huang, J., Lin, Q., Fei, H., He, Z., Xu, H., Li, Y., Qu, K., Han, P., Gao, Q., Li, B., et al. (2023). Discovery of deaminase functions by structure-based protein clustering. Cell *186*, 3182–3195.e14.
33. Xiang, J., Xu, W., Wu, J., Luo, Y., Yang, B., and Chen, J. (2023). Nucleoside deaminases: the key players in base editing toolkit. Biophys. Rep. *9*, 325–337.
34. Deng, J., Li, X., Yu, H., Yang, L., Wang, Z., Yi, W., Liu, Y., Xiao, W., Xiang, H., Xie, Z., et al. (2024). Accelerated discovery and miniaturization of novel single-stranded cytidine deaminases. Nucleic Acids Res. *52*, 11188–11202.
35. Xu, K., Feng, H., Zhang, H., He, C., Kang, H., Yuan, T., Shi, L., Zhou, C., Hua, G., Cao, Y., et al. (2024). Structure-guided discovery of highly efficient cytidine deaminases with sequence-context independence. Nat. Biomed. Eng. *9*, 93–108.
36. Zhang, D., Parth, F., da Silva, L.M., Ha, T.C., Schambach, A., and Boch, J. (2025). Engineering a bacterial toxin deaminase from the DYW-family into a novel cytosine base editor for plants and mammalian cells. Genome Biol. *26*, 18.
37. Yin, L., Shi, K., Chen, Y., Harris, R.S., and Aihara, H. (2024). Structural basis for sequence context-independent single-stranded DNA cytosine deamination by the bacterial toxin SsdA. Preprint at bioRxiv. https://doi.org/10.1101/2024.09.08.611884.
38. Grunewald, J., Zhou, R., Iyer, S., Lareau, C.A., Garcia, S.P., Aryee, M.J., and Joung, J. K. (2019). CRISPR DNA base editors with reduced RNA off-target and self-editing activities. Nat. Biotechnol. *37*, 1041–1048.
39. Sung, Y.H., Kim, J.M., Kim, H.T., Lee, J., Jeon, J., Jin, Y., Choi, J.H., Ban, Y.H., Ha, S. J., Kim, C.H., et al. (2014). Highly efficient gene knockout in mice and zebrafish with RNA-guided endonucleases. Genome Res. *24*, 125–131.

YMTHE, Volume *33*

Supplemental Information

High-efficiency base editing

for nuclear and mitochondrial DNA

with an optimized DYW-like deaminase

Jiyeon Kweon, Soomin Park, Mi Yeon Jeon, Kayeong Lim, Gayoung Jang, An-Hee Jang, Minyoung Lee, Cheong Seok, Chaeyeon Lee, Subin Park, Jiseong Ahn, JiYoon Jang, Naheun Kim, Young Hoon Sung, Daesik Kim, and Yongsub Kim

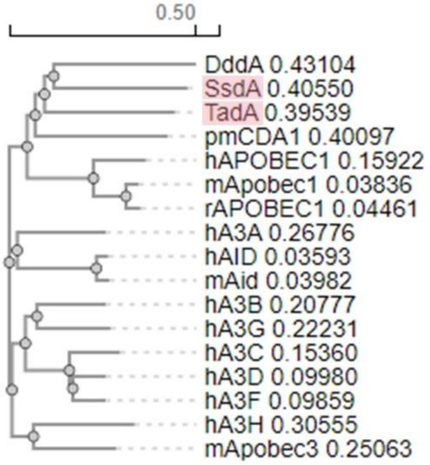

Fig. S1. Phylogenetic tree of representative 18 deaminase domains.

Phylogenetic tree was obtained by T−COFFEE Multiple Sequence Alignment. SsdA and TadA, which served as the basis of rational engineering in this study were highlighted in red.

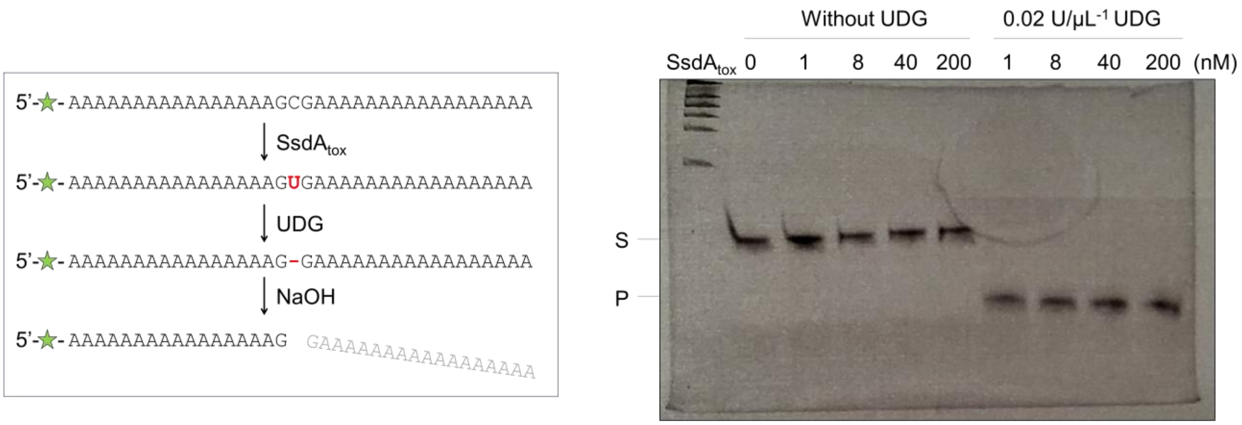

Fig. S2. ***In vitro*** **deaminase assay using SsdA$_{tox}$ protein.**

A 5'-FAM labeled ssDNA containing a single cytosine base nucleotide was used as the substrate for the SsdA$_{tox}$ protein. Cytosine deaminated by the SsdA$_{tox}$ domain is converted to uracil, which is then recognized and cleaved by UDG. NaOH treatment denatures the ssDNA at the cleaved position.

a

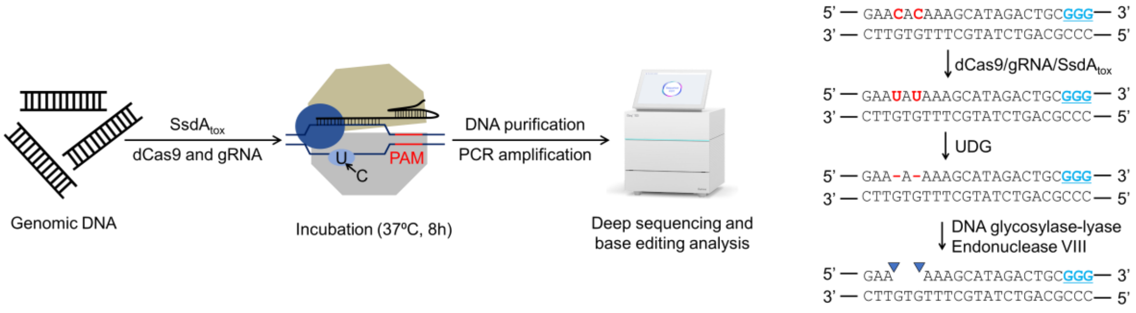

b

RNF2 GTC$_3$ATC$_6$TTAGTC$_{12}$ATTACCTGAGG

USER	-	-	+
RNF2 gRNA	-	+	+
$SsdA_{tox}$/dCas9	-	+	+
C_3	0.1	6.5	0.1
C_6	0.2	0.0	0.0
C_{12}	0.1	10.8	0.0

HEK2 GAAC$_4$AC$_6$AAAGC$_{11}$ATAGACTGCGGG

USER	-	-	+
HEK2 gRNA	-	+	+
$SsdA_{tox}$/dCas9	-	+	+
C_4	0.1	18.5	0.0
C_6	0.0	18.4	0.0
C_{11}	0.1	18.3	0.0

Fig. S3. *In vitro* deaminase assay of $SsdA_{tox}$ with dCas9/gRNA complex.

a Schematic overviews of *in vitro* deaminase assay. Genomic DNA from HEK293T/17 cells were subjected to *in vitro* deaminase assay and cytosine−to−uracil conversion frequencies were measured by targeted deep sequencing. **b** *In vitro* deaminase assay utilizing the $SSdA_{tox}$ domain showed C−to−T conversion efficiencies at the RNF2 (C3, C6, and C12) and HEK2 (C4, C6, and C11) target sites measured by targeted deep sequencing. The maximum conversion efficiency reached up to 50%, limited to editing of cytosine in the target strand only.

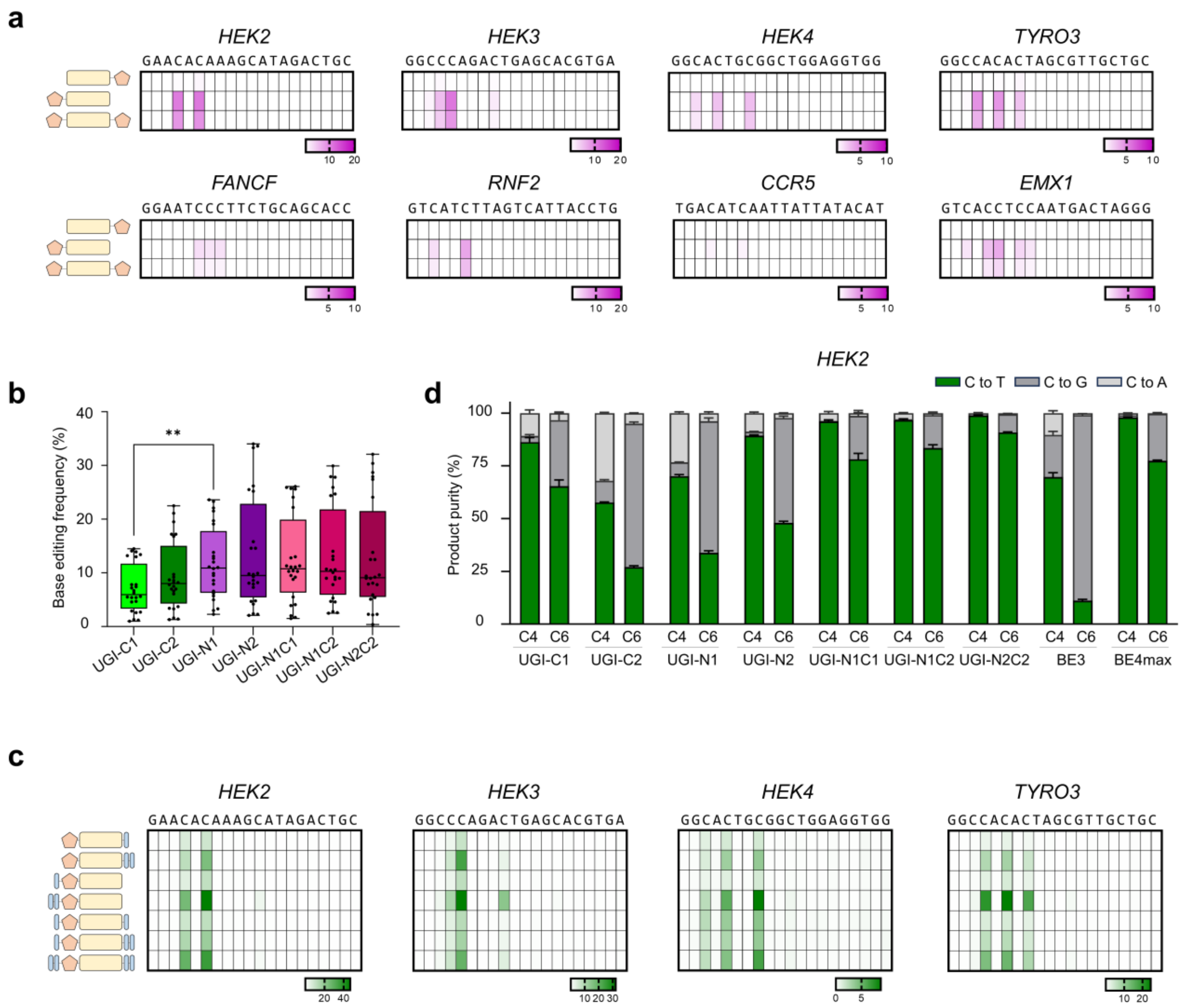

Fig. S4. Developments of $SsdA_{tox}$–based cytosine base editors.

a Base editing frequency of each construct in HEK293T/17 *UNG* KO cells across eight target sites were described in heatmap. **b** Base editing frequencies of each construct across 8 target sites in *UNG* KO HEK293T/17 cells were depicted in box-whisker plot. Dots represent the three independent biological replicates of each 8 target sites. ** P=0.0056 by unpaired t-test. **c** Base editing frequency of each construct in wild-type HEK293T/17 cells across four target sites were described in heatmap. **d** Product purity comparison of each construct at HEK2 C4 and C6 target site in wild-type HEK293T/17 cells. Data are represented as mean of three independent biological replicates (n=3) samples, with error bars representing the SEM.

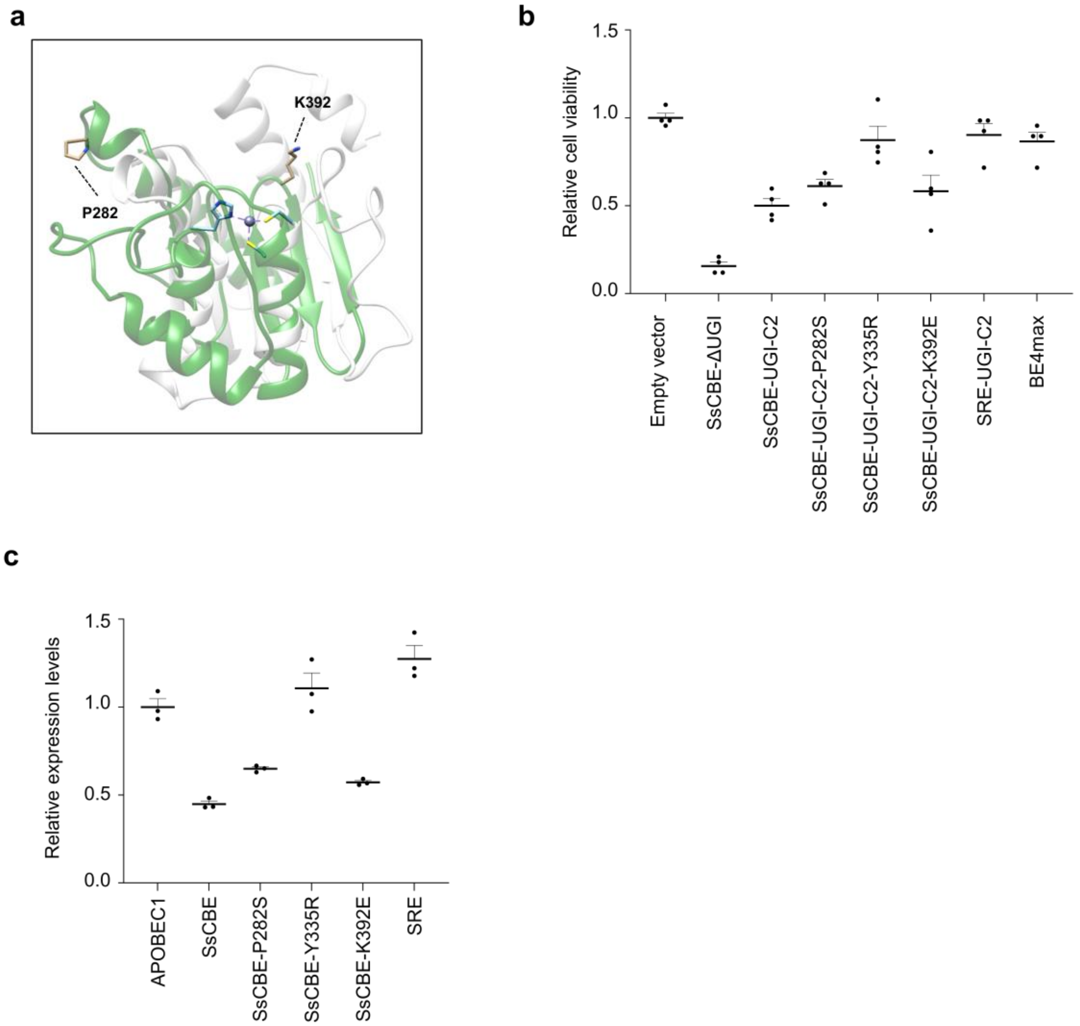

Fig. S5. Cytotoxicity and expression of engineered SsdAtox variants.

a Structural alignment of TadA and SsdA$_{tox}$ domain highlighting two candidate residues for engineering: P282 and K392. **b, c** To measure cell viability, each SsdA$_{tox}$ variant was transfected into HEK293T/17 cells, and the luminescent assay was conducted 72h after transfection. Relative cell viabilities were calculated by dividing the Relative Light Units (RLUs) of cells transfected with each variant by the RLUs of cells transfected with the empty vector. To compare expression level, the P2A-mcherry fused each deaminase domain was transfected into HEK293T/17 cells and FACS analysis was performed. The relative expression levels were calculated by dividing the percentage of PerCP−Cy5−5−A positive cells transfected with each variant by the percentage of PerCP−Cy5−5−A positive cells transfected with the rAPOBEC1−P2A−mcherry construct. The transfection was conducted in three independent biological replicates (n=3).

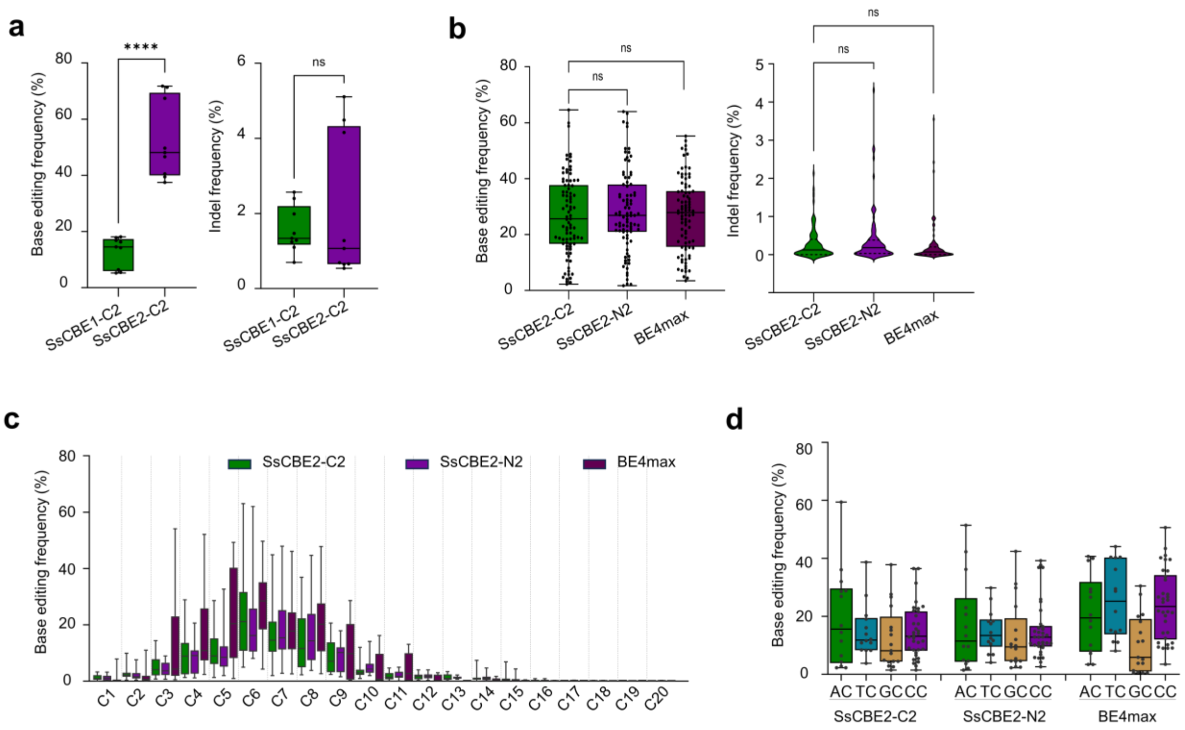

Fig. S6. Rational engineering of SsdA$_{tox}$ domain.

a Comparison of base editing and indel frequency of SsCBE1−C2 and SsCBE2−C2 across three target sites in HEK293T/17 cells. **** P<0.0001 by unpaired t-test. **b** Comparison of base editing and indel frequency of SsCBE2−C2, SsCBE2−N2, and BE4max across 29 endogenous target sites in HEK293T/17 cells. Statistical analysis performed using unpaired t-test. **c, d** Base editing window and context analysis of three constructs across 29 endogenous target sites in HEK293T/17 cells.

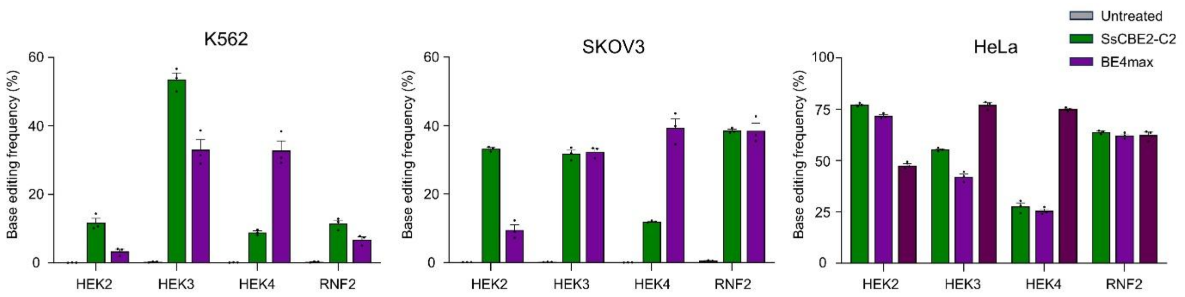

Fig. S7. Base editing in other three cell lines.

SsCBE2-C2 and BE4max were transfected in three different cell line and base editing frequencies were measured by targeted deep sequencing across 4 target sites. Data are presented as mean, with error bars representing SEM of three independent biological replicates (n=3).

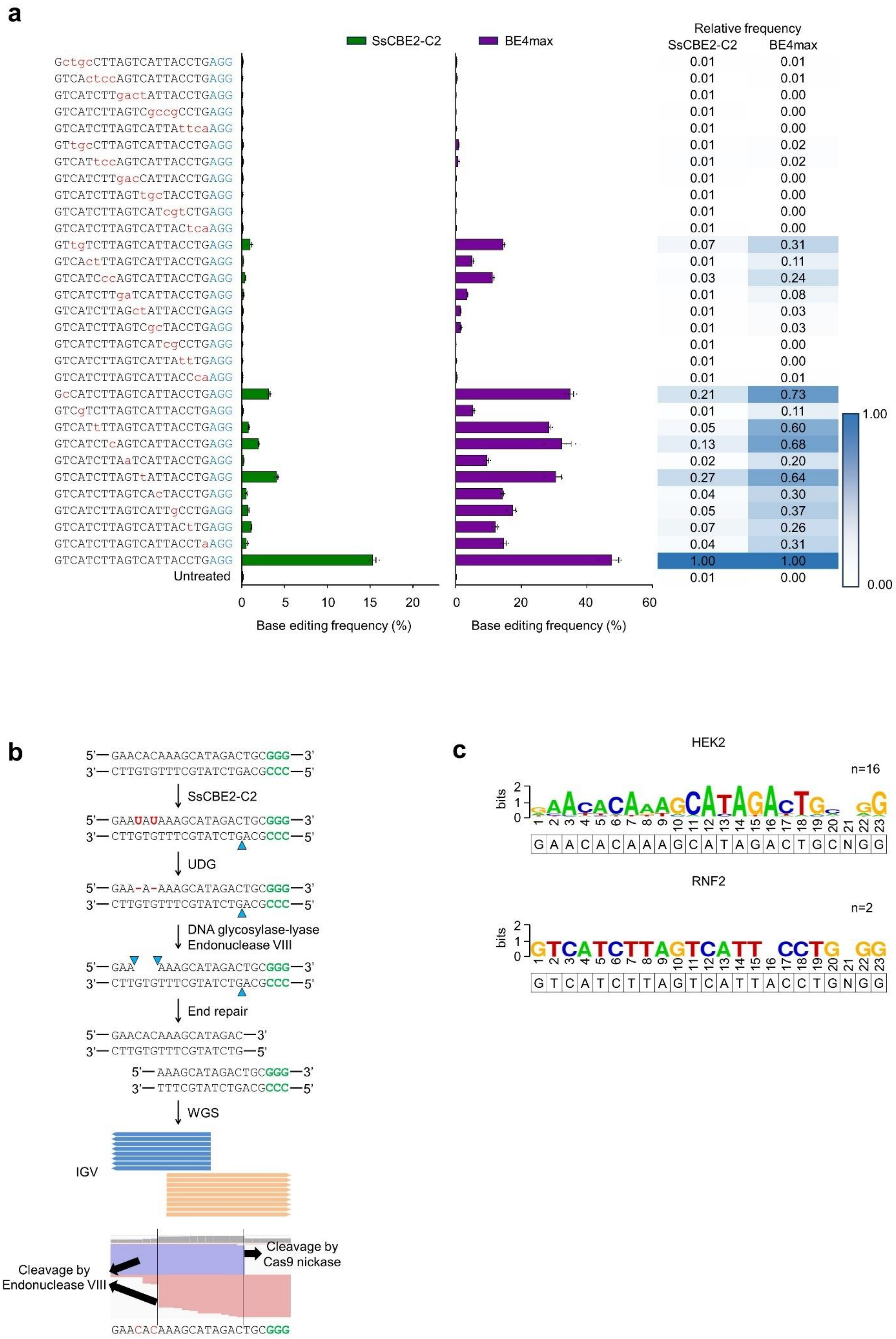

a
SsCBE2-C2
BE4max
Relative frequency
SsCBE2-C2 BE4max
GctgcCTTAGTCATTACCTGAGG 0.01 0.01
GTCActccAGTCATTACCTGAGG 0.01 0.01
GTCATCTTgactATTACCTGAGG 0.01 0.00
GTCATCTTAGTCgccgCCTGAGG 0.01 0.00
GTCATCTTAGTCATTAttcaAGG 0.01 0.00
GTtgcCTTAGTCATTACCTGAGG 0.01 0.02
GTCATtccAGTCATTACCTGAGG 0.01 0.02
GTCATCTTgacCATTACCTGAGG 0.01 0.00
GTCATCTTAGTtgcTACCTGAGG 0.01 0.00
GTCATCTTAGTCATcgtCTGAGG 0.01 0.00
GTCATCTTAGTCATTACtcaAGG 0.01 0.00
GTtgTCTTAGTCATTACCTGAGG 0.07 0.31
GTCActTTAGTCATTACCTGAGG 0.01 0.11
GTCATCccAGTCATTACCTGAGG 0.03 0.24
GTCATCTTgaTCATTACCTGAGG 0.01 0.08
GTCATCTTAGctATTACCTGAGG 0.01 0.03
GTCATCTTAGTCgcTACCTGAGG 0.01 0.03
GTCATCTTAGTCATcgCCTGAGG 0.01 0.00
GTCATCTTAGTCATTAttTGAGG 0.01 0.00
GTCATCTTAGTCATTACCcaAGG 0.01 0.01
GcCATCTTAGTCATTACCTGAGG 0.21 0.73
GTCgTCTTAGTCATTACCTGAGG 0.01 0.11
GTCATtTTAGTCATTACCTGAGG 0.05 0.60
GTCATCTcAGTCATTACCTGAGG 0.13 0.68
GTCATCTTAaTCATTACCTGAGG 0.02 0.20
GTCATCTTAGTtATTACCTGAGG 0.27 0.64
GTCATCTTAGTCAcTACCTGAGG 0.04 0.30
GTCATCTTAGTCATTgCCTGAGG 0.05 0.37
GTCATCTTAGTCATTACtTGAGG 0.07 0.26
GTCATCTTAGTCATTACCTaAGG 0.04 0.31
GTCATCTTAGTCATTACCTGAGG 1.00 1.00
Untreated 0.01 0.00
1.00
0.00
0 5 10 15 20
Base editing frequency (%)
0 20 40 60
Base editing frequency (%)
b
5'— GAACACAAAGCATAGACTGCGGG—3'
3'— CTTGTGTTTCGTATCTGACGCCC—5'
SsCBE2-C2
5'— GAAUAUAAAGCATAGACTGCGGG—3'
3'— CTTGTGTTTCGTATCTGACGCCC—5'
UDG
5'— GAA-A-AAAGCATAGACTGCGGG—3'
3'— CTTGTGTTTCGTATCTGACGCCC—5'
DNA glycosylase-lyase
Endonuclease VIII
5'— GAA AAAGCATAGACTGCGGG—3'
3'— CTTGTGTTTCGTATCTGACGCCC—5'
End repair
5'— GAACACAAAGCATAGAC—3'
3'— CTTGTGTTTCGTATCTG—5'
5'— AAAGCATAGACTGCGGG—3'
3'— TTTCGTATCTGACGCCC—5'
WGS
IGV
Cleavage by Cas9 nickase
Cleavage by Endonuclease VIII
GAACACAAAGCATAGACTGCGGG
c
HEK2
n=16
bits
G A A C A C A A A G C A T A G A C T G C N G G
RNF2
n=2
bits
G T C A T C T T A G T C A T T A C C T G N G G

Fig. S8. Analysis of gRNA dependent DNA off-target effects of SsCBE2.

a Tolerance evaluation of SsCBE2−C2 and BE4max for mismatched sgRNAs with one to four nucleotides mismatches from the RNF2 site in HEK293T/17 cells. PAM sequences are indicated in blue and mismatched bases are indicated in red. Relative frequencies were calculated by dividing base editing frequencies obtained with mismatched sgRNAs by the mean base editing frequency of the matched sgRNA. Data are presented as mean, with error bars representing SEM of three independent biological replicate (n=3). **b** Overviews of Digenome−seq using SsCBE2−C2 at HEK2 target site. SsCBE−C2 catalyzes C−to−U conversion and the uracil−containing sites were cleaved by USER enzyme, a mixture of *E.coli* uracil DNA glycosylase (UDG) and DNA glycosylase−lyase endonuclease VIII. Arrows indicate the positions of phosphodiester bonds cleaved by the SpCas9(D10A) nickase and USER. IGV image shows straight alignments of sequence reads at HEK2 on-target site. **c** Nucleotide sequences captured by Digenome−seq were compared and sequence logos were obtained using WebLogo.

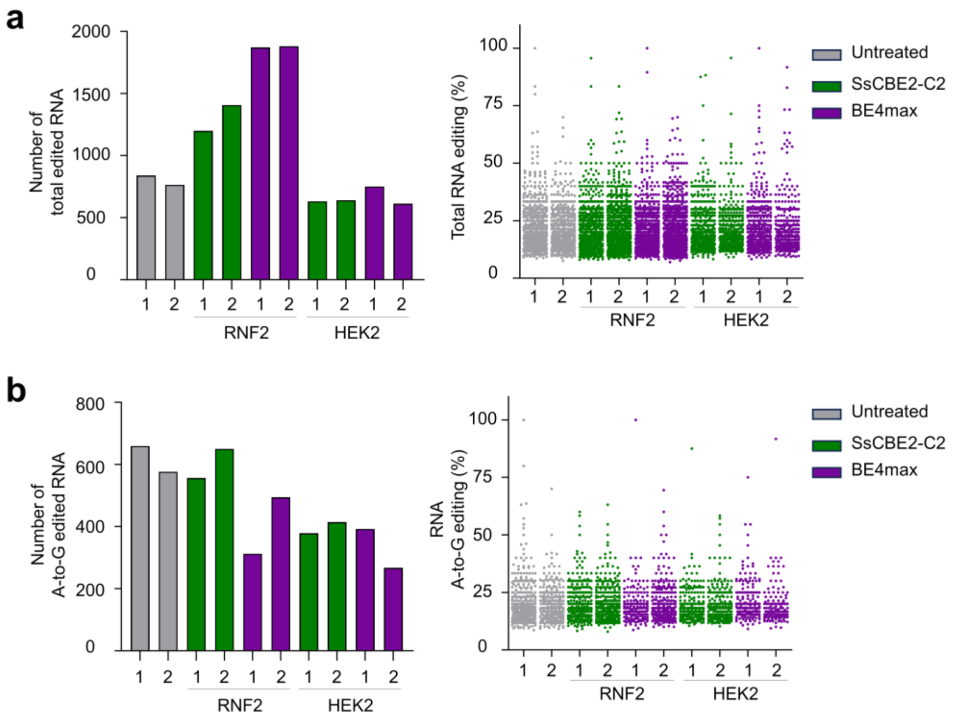

Fig. S9. Analysis of Cas9−independent RNA off−target effects of SsCBE2.

Cas9−independent RNA off−target deamination of SsCBE2−C2 and BE4max in HEK293T/17 cells. Transcriptome sequencing was used to determine the number and frequency of total RNA editing, **a**, and of A−to−G RNA editing, **b**.

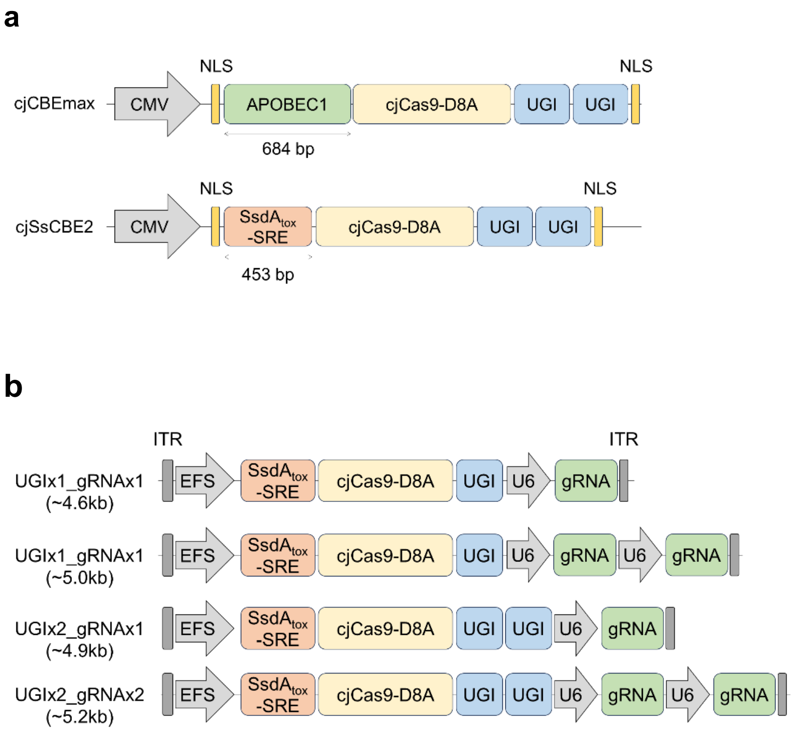

Fig. S10. Architectures of cjCas9-based cytosine base editors.

a Schematic overview of cjSsCBE2 and cjCBEmax. **b** Schematic overviews of single AAV vectors encoding various cjSsCBE2 variants and gRNA. The length between ITR is described in each construct.

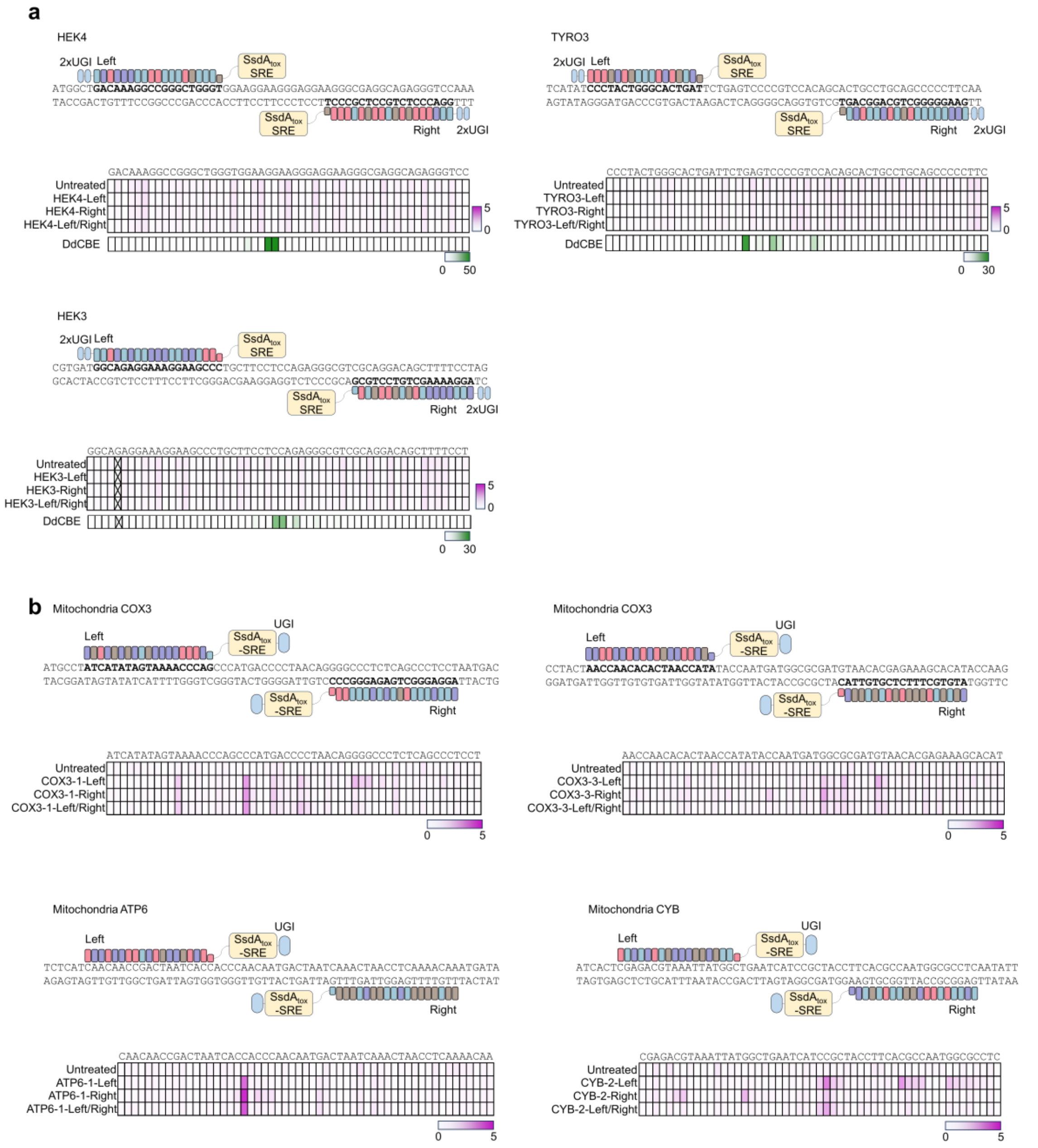

Fig. S11. Base editing using TALE-SRE.

a Base editing frequency of TALE−SRE targeting nucleus genome. Monomer or dimer forms of TALE−SRE were transfected in HEK293T/17 cells and DdCBE was used as positive controls. Transfection was conducted in biological triplicate and base editing frequencies were described in heatmaps. **b** Base editing frequencies induced by TALE–SRE across the target region, including TALE-binding sites and the spacer. Transfection was conducted in biological triplicate and base editing frequencies were described in heatmaps.

Table S1. Identified potential off-target sites by Digenome-seq.

HEK2	Chr.	Location	DNA cleavage score	DNA seq at a cleavage site	Bulge
HEK2_ON	chr.5	87240613	14.2	GAACACAAAGCATAGACTGCGGG	X
HEK2_OT1	chr.15	93557679	7.5	GAACACA-tGCATAGACTGCTAG	O
HEK2_OT2	chr.4	90522183	6.7	GAACACAAtGCATAGAtTGCCGG	X
HEK2_OT3	chr.2	19844956	1.3	aActcCAAAGCATAtACTGCTGG	X
HEK2_OT4	chr.13	55564918	1.2	acACACAAAGCAT-GACTGCAGG	X
HEK2_OT5	chr.1	167742859	0.7	aAACACAgAGCAcAGACTGCTGA	X
HEK2_OT6	chr.19	35505485	0.6	GAACAC-AAGCAcAGACTGaAGG	O
HEK2_OT7	chr.1	36097072	0.4	GtAaACAAAGCATAGACTGaGGG	X
HEK2_OT8	chr.2	192248363	0.4	GAACACAtA-CATAGACaGCTGG	X
HEK2_OT9	chr.11	128508576	0.3	GAAttCAAAGCATAGAtTGCAGG	X
HEK2_OT10	chr.1	77190607	0.3	tcACACAAAcCATAGACTGaGGG	X
HEK2_OT11	chr.4	135329594	0.3	tAACAaAtAGCATAGACTGtGTG	X
HEK2_OT12	chr.8	97317606	0.3	GAACACAgtaCATAGACTGgCAG	X
HEK2_OT13	chr.9	290167	0.2	aAACAtAAAGaATAGACTGCAAG	X
HEK2_OT14	chr.4	53536209	0.2	GAAtACtAAGCATAGACTcCAGG	X
HEK2_OT15	chr.19	28824655	0.2	GAACtCAAAGCATAGAaTaaTGG	X
**RNF2**					
RNF2_ON	chr.1	185056773	3.1	GTCATCTTAGTCATTACCTGAGG	X
RNF2_OT2	chr.10	75832488	0.5	GcCATCTTAGTCATT-CCTGGGG	O

Table S2. Nucleotide sequences of target sites and PCR primers.

Target sites	Spacer sequences	PAM	PCR-F	PCR-R
AAVS1	GCTGACTCAGAGACCCTGAG	TGG	GGCCCAGACTAGCCCAGTTGT	CCACCTGCCTTGGCCTCTCA
CCR5	TGACATCAATTATTATACAT	CGG	GAGGGCAACTAAATACATTCTAGGAC	CCAAAGATGAACACCAGTGA
CUL3	GTAAACCTGGAATAACACGA	TGG	TTGGGAGCACTTCCAGGTTCACT	CTGCACTCCAGCCTTGGTGACAG
EMX1	GTCACCTCCAATGACTAGGG	TGG	GGACAAAGTACAAACGGCAGA	AGTGGCCAGAGTCCAGCTT
FANCF	GGAATCCCTTCTGCAGCACC	TGG	ATGGATGTGGCGCAGGTAG	AGCATTGCAGAGAGGCGTAT
HEK2	GAACACAAAGCATAGACTGC	GGG	AGACCTGGCTGAGCTAACTG	TCCAGCCCCATCTGTCAAAC
HEK3	GGCCCAGACTGAGCACGTGA	TGG	GCATGCATTTGTAGGCTTGA	CCCAGCCAAACTTGTCAAC
HEK4	GGCACTGCGGCTGGAGGTGG	GGG	CTCCCTTCAAGATGGCTGAC	AACGGAGACACACACACAGG
RNF2	GTCATCTTAGTCATTACCTG	AGG	ATTTCCAGCAATGTCTCAGG	GCCAACATACAGAAGTCAGGAA
TYRO3	GGCCACACTAGCGTTGCTGC	TGG	TCCCTACTGGGCACTGATTC	TCCCTGTCAACAAAGTGCTG
Site-1	CCAGCCCGCTGGCCCTGTAA	AGG	AGACCTGGCTGAGCTAACTG	TCCAGCCCCATCTGTCAAAC
Site-2	GCTGGCCCTGTAAAGGAAAC	TGG	AGACCTGGCTGAGCTAACTG	TCCAGCCCCATCTGTCAAAC
Site-3	GTTTCCTTTACAGGGCCAGC	GGG	AGACCTGGCTGAGCTAACTG	TCCAGCCCCATCTGTCAAAC
Site-4	GCACTTGTTTGCAGCTATTC	AGG	AGACCTGGCTGAGCTAACTG	TCCAGCCCCATCTGTCAAAC
Site-5	CTGCTTCTCCAGCCCTGGCC	TGG	GCATGCATTTGTAGGCTTGA	CCCAGCCAAACTTGTCAAC
Site-6	CCCTGGCCTGGGTCAATCCT	TGG	GCATGCATTTGTAGGCTTGA	CCCAGCCAAACTTGTCAAC
Site-7	GGAAGCCCTGCTTCCTCCAG	AGG	GCATGCATTTGTAGGCTTGA	CCCAGCCAAACTTGTCAAC
Site-8	CTTCCTCCAGAGGGCGTCGC	AGG	GCATGCATTTGTAGGCTTGA	CCCAGCCAAACTTGTCAAC
Site-9	CAGGACAGCTTTTCCTAGAC	AGG	GCATGCATTTGTAGGCTTGA	CCCAGCCAAACTTGTCAAC
Site-10	CAGCTCCTGCACCGGGATAC	TGG	GCATGCATTTGTAGGCTTGA	CCCAGCCAAACTTGTCAAC
Site-11	GGGGCACCGCGGCGCCCCGG	TGG	CTCCCTTCAAGATGGCTGAC	AACGGAGACACACACACAGG
Site-12	GCGGCGCCCCGGTGGCACTG	CGG	CTCCCTTCAAGATGGCTGAC	AACGGAGACACACACACAGG
Site-13	CGCCCCGGTGGCACTGCGGC	TGG	CTCCCTTCAAGATGGCTGAC	AACGGAGACACACACACAGG
Site-14	TCCCTTCCTTCCACCCAGCC	CGG	CTCCCTTCAAGATGGCTGAC	AACGGAGACACACACACAGG
Site-15	CCCTGCCTGTCATCCTGCTT	TGG	CTCCCTTCAAGATGGCTGAC	AACGGAGACACACACACAGG
Site-16	GCAGTGCCACCGGGGCGCCG	CGG	CTCCCTTCAAGATGGCTGAC	AACGGAGACACACACACAGG
Site-17	CTCCAGCCGCAGTGCCACCG	GGG	CTCCCTTCAAGATGGCTGAC	AACGGAGACACACACACAGG
Site-18	ACCTCCAGCCGCAGTGCCAC	CGG	CTCCCTTCAAGATGGCTGAC	AACGGAGACACACACACAGG
Site-19	TACACGTCTCATATGCCCCT	TGG	ATTTCCAGCAATGTCTCAGG	GCCAACATACAGAAGTCAGGAA
Site-20	TCAACCATTAAGCAAAACAT	GGG	ATTTCCAGCAATGTCTCAGG	GCCAACATACAGAAGTCAGGAA
Rloop-site-1	GTGGTAGACAGCATGTGTCCTA	AAGGG	TCCTGCAGTCTCCTGCTTCT	ACCAACATACATGCCCCTTT
Rloop-site-2	ATTTACAGCCTGGCCTTTGGGG	TCGGG	GACATTTCCACCGCAAAATG	CGGTGGGAGATCTGGTTTC
Rloop-site-3	GTGTCAGGTAATGTGCTAAACA	GAGAG	TGCTCCAGATTTCCCTTCAT	GGCATCCAGAGACATGGTTT
Rloop-site-4	TCTGCTTCTCCAGCCCTGGC	CTGGG	AAACGCCCATGCAATTAGTC	CAGGAGCTGCACATACTAGCC
Rloop-site-5	GATGTTCCAATCAGTACGCA	GAGAG	GAAAAGCGATCCAGGTGCT	GGCTTTTAAGTTGCCCAGAG
EPAS	CAGGACAGCAGGGGCTCCTTGT	AGCCACAC	AAGCCTTGGAGGGTTTCATT	GTGGCTAGCACCTTCCACTC
HIF	GCTATTCACCAAAGTTGAATCA	GAAGATAC	CCCTTCCCCTCACTGTATCA	GGCCAGCAAAGTTAAAGCAT
ANGPT2	GCTGTGCAGAGGGACGCGCCGC	TCGAATAC	ATGGGTCCTGCAGCTACACT	TTCCATGATGTTCTCCAGCA
HPD-1	TTTTCCACCCGTAGTATGGGGA	CACCACAC	GGAAGTAGGGGGTCCATGA	ACGCATCTGGTTAGGGTCAG
HPD-2	TTCCACCCGTAGTATGGGGACA	CCACACAC	GGAAGTAGGGGGTCCATGA	ACGCATCTGGTTAGGGTCAG
mTyr	ACCTCAGTTCCCCTTCAAAG	GGG	AACCCATGAAGTTGCCTGAG	TTGTTGGCAAAAGAATGCTG

Table S3. Target sequences and PCR sequences for TALE-SRE.

Target sites	Target sequence	PCR-F	PCR-R
ND1-Left	AGCCGTTTACTCAATCCTC	GCTCTCACCATCGCTCTTCT	TGATGGCTAGGGTGACTTCAT
ND1-Right	CAGGGCGTAGTTTGAGTTT	GCTCTCACCATCGCTCTTCT	TGATGGCTAGGGTGACTTCAT
ATP6-1-Left	CAACAACCGACTAATCACC	CCCTCTATTGATCCCCACCT	GATGGCCATGGCTAGGTTTA
ATP6-2-Right	TTGTTTTGAGGTTAGTTTG	CCCTCTATTGATCCCCACCT	GATGGCCATGGCTAGGTTTA
ATP6-2-Left	AACCATACACAACACTAAA	CCCTCTATTGATCCCCACCT	GATGGCCATGGCTAGGTTTA
ATP6-2-Right	GATTAAGGATACTAGTATA	CCCTCTATTGATCCCCACCT	GATGGCCATGGCTAGGTTTA
COX3-1-Left	ATCATATAGTAAAACCCAG	CAACACATAATGACCCACCAA	GAAGGCCTTTTTGGACAGGT
COX3-1-Right	AGGAGGGCTGAGAGGGCCC	CAACACATAATGACCCACCAA	GAAGGCCTTTTTGGACAGGT
COX3-2-Left	CCTAATGACCTCCGGCCTA	CAACACATAATGACCCACCAA	GAAGGCCTTTTTGGACAGGT
COX3-2-Right	GAGGAGCGTTATGGAGTGG	CAACACATAATGACCCACCAA	GAAGGCCTTTTTGGACAGGT
COX3-3-Left	AACCAACACACTAACCATA	CAACACATAATGACCCACCAA	GAAGGCCTTTTTGGACAGGT
COX3-3-Right	ATGTGCTTTCTCGTGTTAC	CAACACATAATGACCCACCAA	GAAGGCCTTTTTGGACAGGT
CYB-1-Left	CCAACATCTCCGCAT	AACCACTCATTCATCGACCTC	CGCCCGATGTGTAGGAAG
CYB-1-Right	CAGGCAGGCGCCAAGGAGT	AACCACTCATTCATCGACCTC	CGCCCGATGTGTAGGAAG
CYB-2-Left	CGAGACGTAAATTATGGC	AACCACTCATTCATCGACCTC	CGCCCGATGTGTAGGAAG
CYB-2-Right	GAGGCGCCATTGGCGTGAA	AACCACTCATTCATCGACCTC	CGCCCGATGTGTAGGAAG
HEK4-Left	GACAAAGGCCGGGCTGGGT	CTCCCTTCAAGATGGCTGAC	AACGGAGACACACACACAGG
HEK4-Right	GGACCCTCTGCCTCGCCCT	CTCCCTTCAAGATGGCTGAC	AACGGAGACACACACACAGG
TYRO3-Left	CCCTACTGGGCACTGAT	TCCCTACTGGGCACTGATTC	TCCCTGTCAACAAAGTGCTG
TYRO3-Right	GAAGGGGGCTGCAGGCAGT	TCCCTACTGGGCACTGATTC	TCCCTGTCAACAAAGTGCTG
HEK3-Left	GGCAGAGGAAAGGAAGCCC	GCATGCATTTGTAGGCTTGA	CCCAGCCAAACTTGTCAAC
HEK3-Right	AGGAAAAGCTGTCCTGCG	GCATGCATTTGTAGGCTTGA	CCCAGCCAAACTTGTCAAC
